# Solid Polymer Electrolytes for Advanced Lithium Batteries: Decoupling Ion Transport, Engineering Interfaces, and Enabling Sustainable Manufacturing

**DOI:** 10.1002/smsc.70403

**Published:** 2026-09-16

**Authors:** Mohan Raj Krishnan, Chandra Sekhar Bongu, Edreese Alsharaeh

**Affiliations:** ^1^ College of Science and General Studies Alfaisal University Riyadh Saudi Arabia

**Keywords:** ion transport mechanisms, polymer–ceramic nanocomposites, solid polymer electrolytes, solid‐state batteries, sustainable electrolyte materials

## Abstract

Solid polymer electrolytes (SPEs) are promising materials for next‐generation lithium batteries owing to their inherent safety, mechanical flexibility, and compatibility with advanced cell architectures. However, their practical implementation remains constrained by low room‐temperature ionic conductivity, solid–solid interfacial resistance, and limited manufacturing scalability. This review critically examines recent advances in SPEs, with particular emphasis on fundamental Li^+^ transport mechanisms and the molecular factors governing ionic mobility, as well as strategies for decoupling ion transport from polymer segmental dynamics. Emerging design strategies, including architecture‐engineered polymers, single‐ion conductors, self‐healing networks, and polymer–ceramic composites, are evaluated in terms of their ability to balance ionic conductivity, mechanical integrity, and interfacial stability. Particular attention is devoted to interfacial phenomena and mesoscale structural regulation that govern practical electrochemical performance. Recent progress in sustainable electrolytes, including fluorine‐free salts, bio‐derived polymers, and recyclable dynamic networks, is also highlighted. Finally, remaining challenges and future opportunities are discussed, emphasizing integrated molecular and interfacial design, standardized performance evaluation, and scalable manufacturing toward safe, sustainable, and high‐performance solid‐state batteries.

## Graphical abstract

Solid polymer electrolytes provide a promising route toward safer, lightweight, and high‐energy solid‐state batteries. This review critically connects ion‐transport mechanisms, interfacial engineering, polymer and composite design, and practical performance, while highlighting persistent conductivity, interface, and scalability challenges. Sustainable materials, fluorine‐free chemistries, and eco‐friendly manufacturing are identified as important pathways toward practical solid‐state batteries.

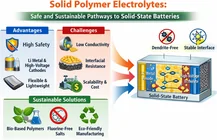

## 1 Introduction

The rapid evolution of portable electronics, electric vehicles (EVs), and renewable energy systems has intensified the global pursuit of high‐performance, safe, and sustainable energy storage technologies [1, 2, 3]. Lithium‐ion batteries (LIBs) have long dominated this landscape due to their high energy density and efficiency [4]. However, conventional liquid electrolytes—typically composed of organic carbonate solvents and lithium salts—pose intrinsic limitations, including flammability, leakage risk, and limited electrochemical stability [5, 6]. These safety and performance constraints have prompted a paradigm shift toward the development of solid‐state electrolytes (SSEs), with solid polymer electrolytes (SPEs) emerging as one of the most promising candidates [7, 8, 9]. SPEs offer a unique combination of mechanical flexibility, lightweight architecture, and compatibility with scalable fabrication techniques. Unlike liquid systems, they provide a leak‐proof, thermally stable, and flame‐retardant medium for ion conduction, significantly enhancing the overall safety of electrochemical devices [10, 11]. The most extensively studied SPEs are based on poly(ethylene oxide) (PEO), first introduced by Armand in 1973 as a lithium‐ion conducting matrix [12]. Since then, numerous polymer systems, such as polyacrylonitrile (PAN) [13, 14], poly(vinylidene fluoride) (PVDF) [15, 16], and poly(methyl methacrylate) (PMMA) [17, 18], have been explored for their distinct chemical and physical characteristics. Ion transport in SPEs primarily relies on the segmental motion of polymer chains, where lithium ions coordinate with electron‐rich atoms (e.g., oxygen or nitrogen) in the polymer backbone [19]. However, this mechanism is inherently coupled with polymer mobility, resulting in a trade‐off between mechanical strength and ionic conductivity [20]. Typical ionic conductivities of conventional SPEs remain in the range of 10^−5^ to 10^−7^ S/cm at room temperature—insufficient for practical applications [21, 22]. To address these challenges, extensive efforts have been directed toward polymer blending, copolymerization, plasticization, and nanofiller incorporation, aiming to enhance both conductivity and structural integrity [23, 24, 25, 26].

**FIGURE 1:**
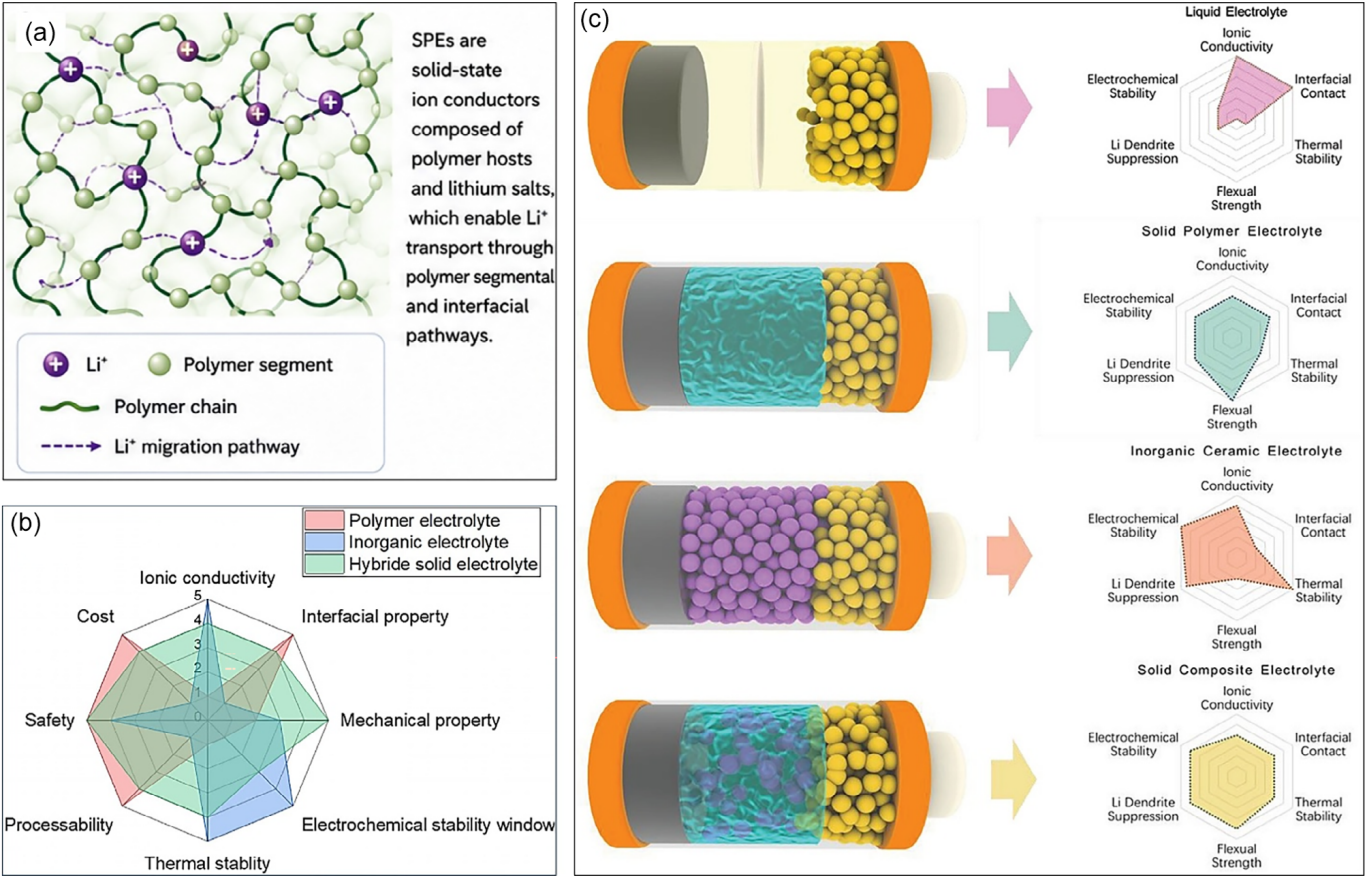
(a) Solid polymer electrolytes for safe and sustainable high‐energy density batteries. (b) Property ranking of polymer electrolytes in comparison to inorganic and solid hybrid electrolytes (Best = 5; worst = 1), Reproduced with permission from ref. [46]. Copyright 2020, Frontiers, and (c) performance comparisons of conventional liquid, polymer, inorganic, and hybrid solid electrolytes, Reproduced with permission from ref. [47]. Copyright 2020, Wiley.

Recent advances in rechargeable battery research have further demonstrated that electrolyte development must be considered alongside innovations in electrode materials and interfacial engineering. For example, regulating the electrostatic microenvironment through anion‐vacancy engineering has been shown to homogenize lithium‐ion distribution and reduce concentration gradients in polymer electrolytes, thereby improving ion‐transport kinetics [27]. Similarly, structural modification of Na‐deficient layered cathodes through lithium incorporation has significantly enhanced high‐voltage cycling stability, highlighting the importance of synergistic electrolyte–electrode design [28]. Considerable progress has also been made in developing nonflammable electrolytes for lithium‐, sodium‐, and potassium‐ion batteries (PIBs) to improve battery safety under practical operating conditions [29]. Beyond conventional lithium‐ion systems, advanced interfacial catalysts and electrocatalytic architectures have enabled more efficient sulfur conversion and improved cycling stability in high‐energy lithium–sulfur batteries, further emphasizing the critical role of electrolyte and interface engineering in next‐generation electrochemical energy storage [30, 31]. In recent years, polymer–ceramic composite electrolytes [32, 33, 34, 35] and single‐ion conducting systems [36, 37, 38, 39] have gained significant attention due to their potential to combine high ionic mobility with mechanical robustness. The integration of inorganic nanoparticles, such as Al_2_O_3_ [40], SiO_2_ [41], and TiO_2_ [42], disrupts polymer crystallinity, facilitates ion dissociation, and strengthens the polymer matrix [43]. Concurrently, advances in molecular engineering have enabled the design of tailored polymer architectures—such as block copolymers and cross‐linked networks—that decouple mechanical and transport properties [44, 45]. Ultimately, the successful development of high‐performance SPEs is anticipated to revolutionize solid‐state battery (SSB) technology, unlocking new frontiers in energy density, safety, and longevity. This work explores the scientific principles, material innovations, and practical challenges associated with SPEs, elucidating their critical role in shaping the next generation of sustainable electrochemical energy systems (Figure 1). Several excellent reviews have recently summarized specific aspects of SPEs, including polymer–electrode interface engineering, composite electrolytes, and individual polymer systems [8, 48, 49, 50]. In particular, Li et al. comprehensively reviewed interfacial design strategies for SPEs, with emphasis on electrode–electrolyte interfaces and their influence on electrochemical performance [51]. In contrast, the present review adopts a broader perspective by integrating the fundamental mechanisms of lithium‐ion transport with recent advances in polymer molecular design, polymer–ceramic composites, block copolymers, single‐ion conductors, self‐healing materials, sustainable manufacturing, and emerging applications. Rather than treating these topics independently, the review highlights the interdependence between molecular architecture, interfacial chemistry, processing strategies, and device‐level performance, thereby providing a comprehensive framework for the rational design and practical implementation of next‐generation SPEs.

## 2 Fundamentals of Lithium‐Ion Transport in SPEs

### 2.1 Polymer Segmental Dynamics and Li^+^ Hopping; and Salt Dissociation and Ion Coordination

**FIGURE 2:**
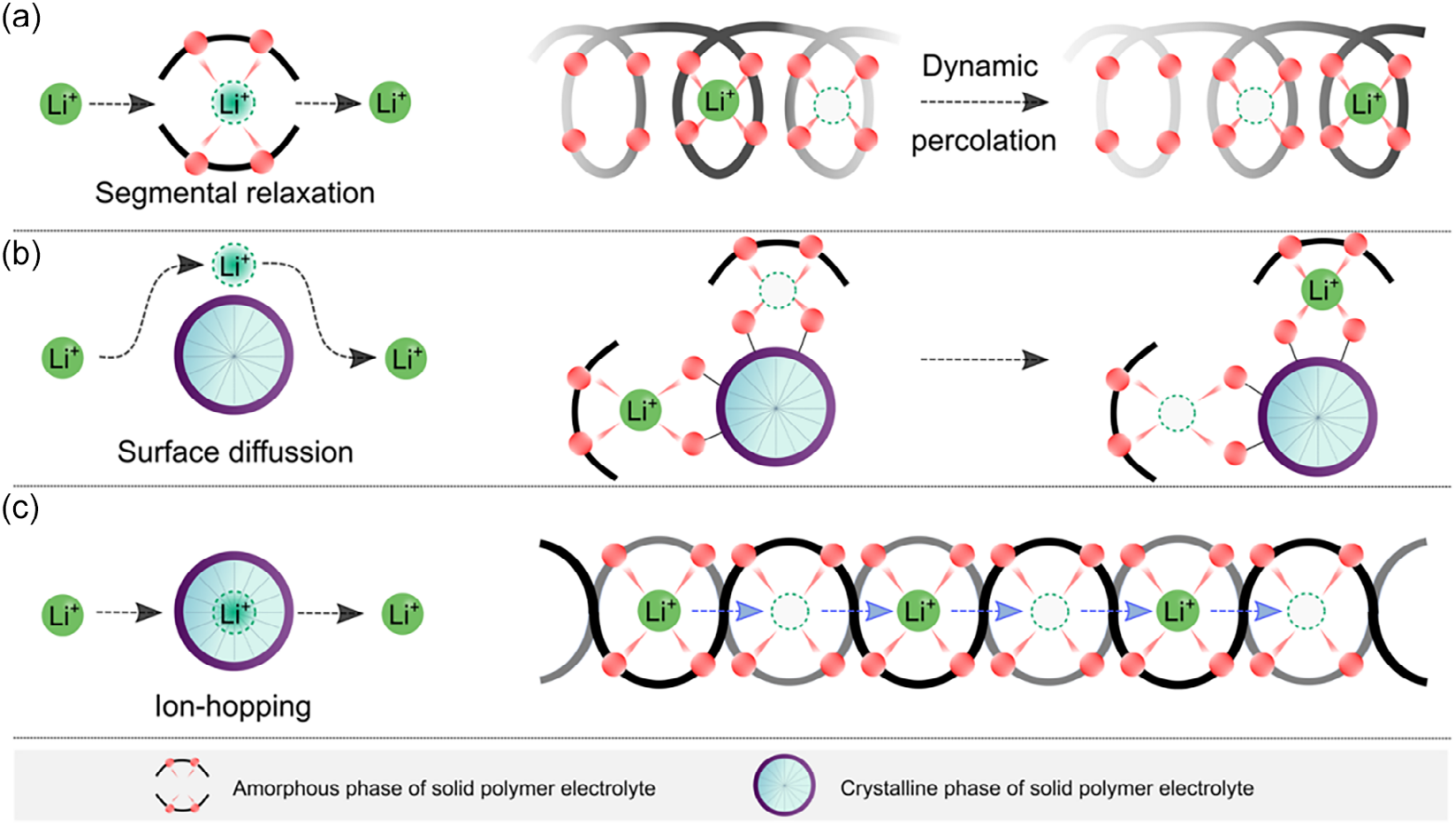
(a) Li^+^ transportation in fully amorphous phases of polymers through segmental relaxation. (b) Li^+^ transportation through mixed amorphous and crystalline phases of polymers, wherein surface functional groups facilitate the ionic transportation in the crystalline phases, and (c) Li^+^ transportation through crystalline phases via a hopping mechanism. Reproduced with permission from ref. [52]. Copyright 2023, Springer Nature.

Ion transport in SPEs is predominantly governed by a polymer segmental‐assisted conduction mechanism, which is fundamentally distinct from the solvated diffusion observed in liquid electrolytes. For early‐developed polyethylene oxide (PEO)‐based SPEs, the Li^+^ conduction mechanisms are summarized in Figure 2 [52]. In predominantly amorphous regions, long‐range transport of Li^+^ is generally governed by the segmental motion of the polymer backbone (Figure 2a). This transport behavior is commonly described by the percolation model, in which ion migration is coupled to local chain dynamics [53]. In systems containing both amorphous and crystalline phases, the crystalline domains influence Li^+^ transport in a more complex manner. While the surfaces of crystalline regions can participate in Li^+^ transport through surface coordination effects (Figure 2b), the crystalline interiors typically do not support ionic motion [54]. As a result, most crystalline PEO–salt complexes exhibit poor ionic conductivity. The formation of crystalline spherulites—characteristic morphological features of crystalline PEO—can therefore significantly reduce overall ionic conductivity by interrupting continuous conduction pathways within the amorphous phase. An exception occurs in well‐defined crystalline complexes, such as (PEO)_6_ lithium hexafluoroarsenate, where ion transport can proceed via lithium‐ion hopping between adjacent coordination sites within the crystal lattice (Figure 2c) [55]. In such systems, the molecular weight of PEO and the nature of the lithium salt play a decisive role in determining transport efficiency as the availability of defects and accessible hopping sites within the crystalline framework strongly influences lithium‐ion mobility.

**FIGURE 3:**
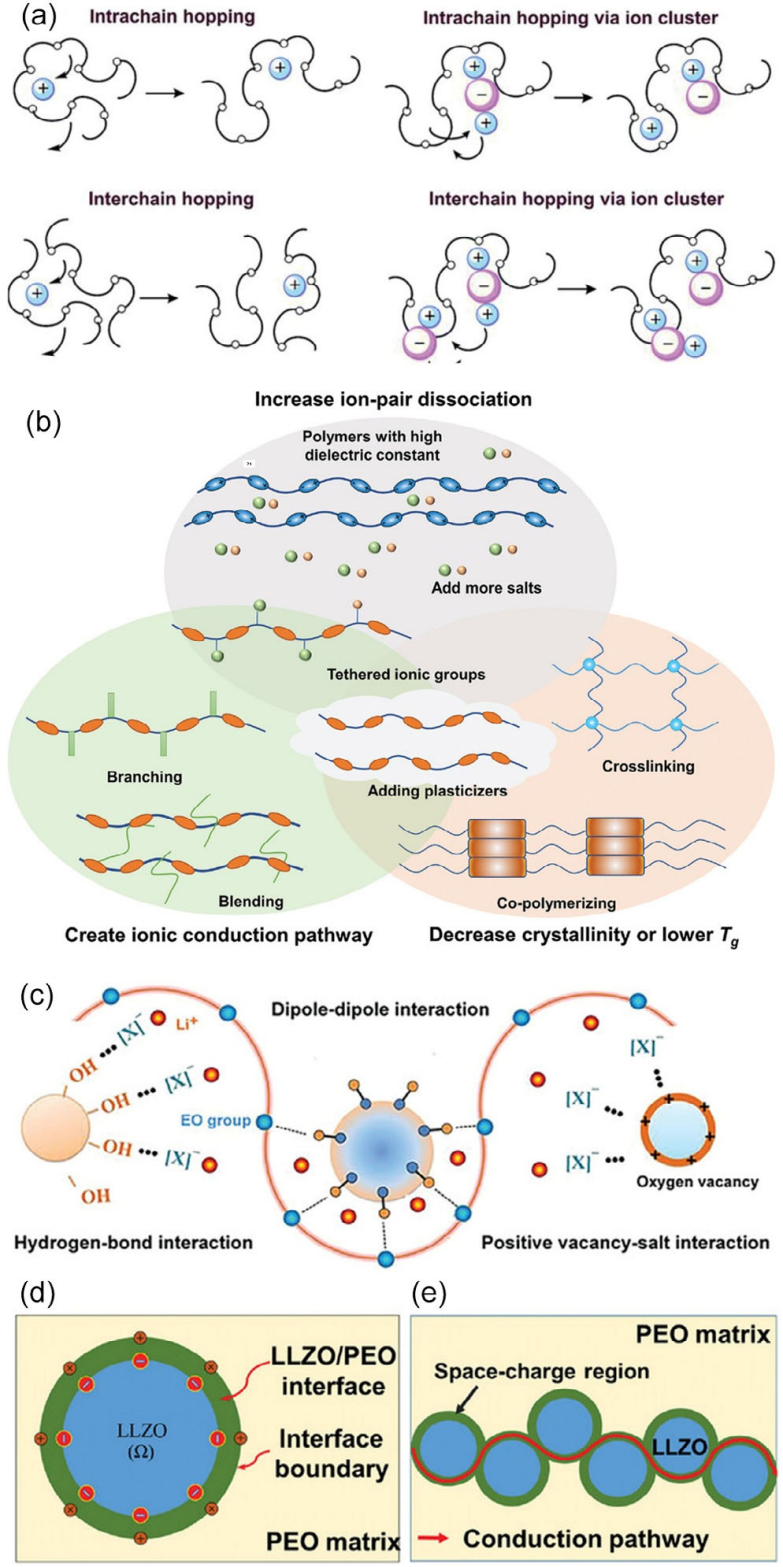
The mechanism of Li^+^ ion transport in SPEs through segmental motion of the polymers and ionic coordination with electronegative atoms. (a) Li^+^ conduction in the amorphous region of the polymer. (b) Various strategies to prepare highly ionic‐conducting SPEs. (c) Lewis acid–base interactions between fillers and polymer. (d) Space‐charge region between polymer and filler, and (e) Space‐charge regions facilitate fast ionic conduction. Reproduced with permission from ref. [56]. Copyright 2023, Wiley.

In general, Li^+^ ions migrate by forming transient coordination complexes with electronegative atoms—typically oxygen or nitrogen—distributed along the polymer backbone (Figure 3) [56, 57]. These weak, reversible interactions allow ions to hop between neighboring coordination sites as the host polymer undergoes segmental motion [58, 59]. As a result, ionic conductivity is intrinsically linked to polymer relaxation dynamics and is strongly influenced by chain flexibility, glass‐transition temperature (T_g_), and amorphous content. The temperature dependence of this transport behavior is often described by non‐Arrhenius models, such as the Vogel–Tamman–Fulcher formalism [60, 61], which highlights the coupled nature of ionic mobility and polymer dynamics. The coordination between lithium ions and polymer functional groups dictates solvation strength and ion dissociation. Polymers containing ether oxygen atoms, such as PEO, exhibit excellent solvating power for lithium salts such as LiClO_4_, LiTFSI, and LiBF_4_ [62]. However, excessive coordination can lead to ion pairing or clustering, reducing the number of free charge carriers. Optimizing salt concentration and polymer–ion interactions is therefore essential to maximize conductivity while maintaining mechanical integrity [63].

Extensive studies indicate that enhancing segmental mobility increases the rate of Li^+^ hopping, whereas crystalline domains restrict chain motion and impede ionic conduction. Consequently, material strategies—including polymer design, incorporation of plasticizers, and addition of ceramic nanofillers—have been employed to suppress crystallinity and stabilize amorphous phases. The coordination strength between Li^+^ and polymer functional groups, as well as the dielectric properties governing salt dissociation, further modulate the efficiency of ion transport. Taken together, these mechanistic insights establish a clear design principle: optimizing the interplay between polymer architecture, segmental mobility, and ion–polymer interactions is essential to achieving high ionic conductivity without sacrificing mechanical integrity. Recent advances in polymer–ceramic nanocomposite electrolytes have demonstrated significant improvements in both conductivity and stability of SPEs [63]. Inorganic fillers such as Al_2_O_3_, SiO_2_, and TiO_2_ introduce Lewis acid–base interactions that promote ion dissociation and disrupt polymer crystallinity [33, 64, 65]. Additionally, these ceramic fillers create fast‐ion transport channels and enhance mechanical rigidity, suppressing dendrite growth in lithium‐metal systems. Interfacial phenomena also play a pivotal role [66, 67]. The polymer–electrode interface must enable efficient ion transfer without forming resistive layers [68]. Surface modifications, such as ionic liquid incorporation [69, 70] or in situ polymerization [71, 72, 73], have been shown to lower interfacial resistance and improve electrode compatibility. Recently, Li et al. provided a comprehensive summary of ionic conduction mechanisms in SPEs, including solvent‐free polymer electrolytes, composite polymer electrolytes, and quasisolid/gel polymer electrolytes [56]. Looking ahead, advancing SPEs for SSB applications will require innovation that transcends traditional approaches. Promising directions include decoupling ion transport from polymer segmental motion via block copolymer architectures, single‐ion conducting frameworks, and polymer–ceramic composite networks. Such strategies aim to enable fast ion conduction while maintaining robustness and electrochemical stability—key prerequisites for realizing safe, sustainable, and high‐energy solid‐state batteries. Decoupling ion transport from polymer segmental relaxation is fundamentally a process of structure‐regulated ion transport, in which the local chemical environment and polymer architecture determine lithium‐ion migration independently of bulk chain dynamics. Beyond improving ionic conductivity, this strategy is closely related to achieving rapid charge storage because the local coordination environment governs ion desolvation, hopping frequency, and transport across heterogeneous interfaces. Recent studies have demonstrated that controlling local structural features—including ion‐conduction channels, coordination environments, and interfacial chemistry—can substantially reduce kinetic limitations and improve high‐rate alkali‐ion storage by facilitating faster ion migration and minimizing concentration polarization [74, 75]. Similar concepts have been reported for advanced alkali‐ion batteries, where local structural regulation directly influences charge‐storage kinetics and electrochemical performance, highlighting the importance of linking atomistic transport mechanisms with device‐level rate capability [76, 77, 78].

### 2.2 Effect of Additive Polarity on Li^+^ Transport

At the same time, the polarity of electrolyte additives plays a critical role in determining lithium‐ion transport in SPEs because it directly influences lithium‐salt dissociation, ion coordination, and polymer segmental dynamics [58]. Additives possessing high dielectric constants increase the polarity of the local polymer environment, thereby reducing electrostatic interactions between lithium cations and their counter anions [9]. Consequently, a greater fraction of free Li^+^ ions becomes available for migration, leading to improved ionic conductivity [79]. Polar organic plasticizers such as succinonitrile, ethylene carbonate, propylene carbonate, and polyethylene glycol derivatives have been widely employed to enhance ion transport by increasing salt dissociation while simultaneously reducing polymer crystallinity [80, 81, 82]. Likewise, ionic liquids containing highly polar functional groups facilitate Li^+^ mobility through weak ion pairing and improved electrochemical stability [83, 84]. However, excessive plasticization may decrease the mechanical strength of the electrolyte, making it more susceptible to lithium dendrite penetration. The polarity of inorganic fillers also contributes to lithium‐ion transport. Ceramic particles with Lewis acidic or Lewis basic surface sites, including Al_2_O_3_, TiO_2_, SiO_2_, LATP, and LLZO, interact with lithium salts and polymer chains, promoting salt dissociation and reducing ion aggregation [85]. Surface functionalization further enhances these interactions by tailoring the interfacial polarity between the ceramic and polymer phases, thereby creating more continuous lithium‐ion migration pathways [86]. Functional additives containing carbonyl, nitrile, sulfone, phosphate, ether, or fluorinated groups modify the local coordination environment surrounding Li^+^ ions [87, 88]. These interactions weaken Li^+^–anion binding, lower the activation energy for ion hopping, and improve ionic conductivity while simultaneously stabilizing the electrode–electrolyte interface. Nevertheless, the relationship between additive polarity and electrochemical performance is not linear. Highly polar additives may improve conductivity but can also increase electrolyte softness, alter oxidative stability, or induce undesirable side reactions if incorporated in excessive amounts. Consequently, optimizing additive polarity requires balancing ionic conductivity, mechanical robustness, electrochemical stability, and long‐term cycling performance. Therefore, additive polarity should be considered an important design parameter in the development of advanced SPEs, as it governs both bulk ion transport and interfacial electrochemical processes.

### 2.3 Role of Interfaces in Li^+^ Transport

Although the incorporation of fillers or multiple phases increases the number of interfaces within composite solid electrolytes, these interfaces do not necessarily impede lithium‐ion transport. Instead, their influence depends primarily on their chemical composition, structural continuity, and interfacial compatibility rather than just their numbers [89]. Poorly bonded interfaces containing voids, impurities, or unstable interphases can act as transport barriers by increasing interfacial resistance and interrupting continuous ion‐conduction pathways. In contrast, chemically engineered interfaces can significantly enhance ionic transport by providing energetically favorable pathways for Li^+^ migration [90].

### 2.4 Advanced Spectroscopic and Computational Insights into Interfacial Ion Transport

Understanding ion transport in solid polymer and polymer–ceramic composite electrolytes requires methods capable of resolving local coordination environments, molecular relaxation, and ion exchange across heterogeneous interfaces. Conventional impedance measurements provide macroscopic conductivity but cannot identify whether Li^+^ migrates predominantly through the polymer phase, ceramic phase, or interfacial regions. Solid‐state nuclear magnetic resonance spectroscopy is particularly valuable in this regard because it can distinguish chemically different Li populations and probe exchange between them. Multidimensional solid‐state NMR measurements on PEO/LiTFSI–LLZO composites have directly demonstrated Li^+^ exchange between polymer‐rich and ceramic‐rich environments while also revealing that surface species such as LiOH can influence the exchange process. These findings show that the ceramic–polymer interface is not necessarily a passive boundary and may participate directly in long‐range Li^+^ transport [91]. NMR studies of hybrid polymer–inorganic electrolytes further demonstrate that interfacial transport depends strongly on local surface chemistry. In PEO‐based hybrid electrolytes containing inorganic conductors and ionic‐liquid interfacial modifiers, solid‐state NMR revealed that improved interfacial wetting and increased local polarizability lowered the Li^+^ diffusional barrier [91]. The resulting enhancement arose from faster exchange across the organic–inorganic interface rather than from a simple increase in ceramic loading [92]. These observations support an interface‐specific design principle: the effectiveness of a filler depends on whether its surface establishes dynamically connected coordination environments with the surrounding polymer.

Quasielastic neutron scattering (QENS) provides complementary dynamic information because it probes polymer motions over picosecond‐to‐nanosecond timescales and ångström‐to‐nanometre length scales. QENS measurements have shown that high polymer polarity does not automatically result in faster ion transport. For example, although nitrile‐containing polymers can promote greater salt dissociation than PEO, their larger monomeric friction and slower segmental relaxation may offset this benefit [93]. More recently, combined QENS and molecular simulations directly identified nanosecond‐scale breakup and reformation of Li^+^ solvation structures in polymer electrolytes [75]. These temporary Li^+^‐mediated crosslinks govern the timescale of solvation‐shell relaxation and therefore influence the rate at which Li^+^ can exchange coordination sites [75]. Such results demonstrate that conductivity is controlled by both the number of dissociated charge carriers and the dynamics of coordination–decoordination events. Atomistic simulations provide further insight into processes that are difficult to isolate experimentally. Classical molecular dynamics (MD) simulations of PEO/LiTFSI in contact with lithium metal have shown that the metal surface alters Li^+^ coordination, polymer orientation, and ion mobility near the interface [94]. More recent density functional theory (DFT) and ab initio molecular dynamics (AIMD) studies have examined the early decomposition of polymer‐electrolyte molecules on Li metal, identifying reaction pathways that precede formation of the solid–electrolyte interphase [95]. These calculations help distinguish polymer structures that form stable passivating products from those that undergo continuous decomposition and generate resistive interfaces. AIMD can also determine preferred coordination geometries, migration pathways, and activation barriers; however, its accessible length and time scales are generally limited to capture the full evolution of heterogeneous SEI or CEI layers. Recent machine‐learning‐assisted studies have reproduced the structural evolution and reaction products of Li‐metal/solid–electrolyte interfaces over hundreds of picoseconds, allowing reaction kinetics and passivation‐layer formation to be compared across different interface chemistries [96]. Machine‐learning potentials have also been applied to SEI materials to investigate Li^+^ diffusion, structural disorder, and dendrite initiation at dimensions inaccessible to conventional AIMD [97]. Nevertheless, their predictive reliability depends strongly on the diversity and quality of the training configurations, particularly when bond breaking, charge transfer, and previously unseen interface structures are involved.

## 3 Solid Electrolytes—Recent Innovations and Emerging Design Paradigms

### 3.1 SPEs

**FIGURE 4:**
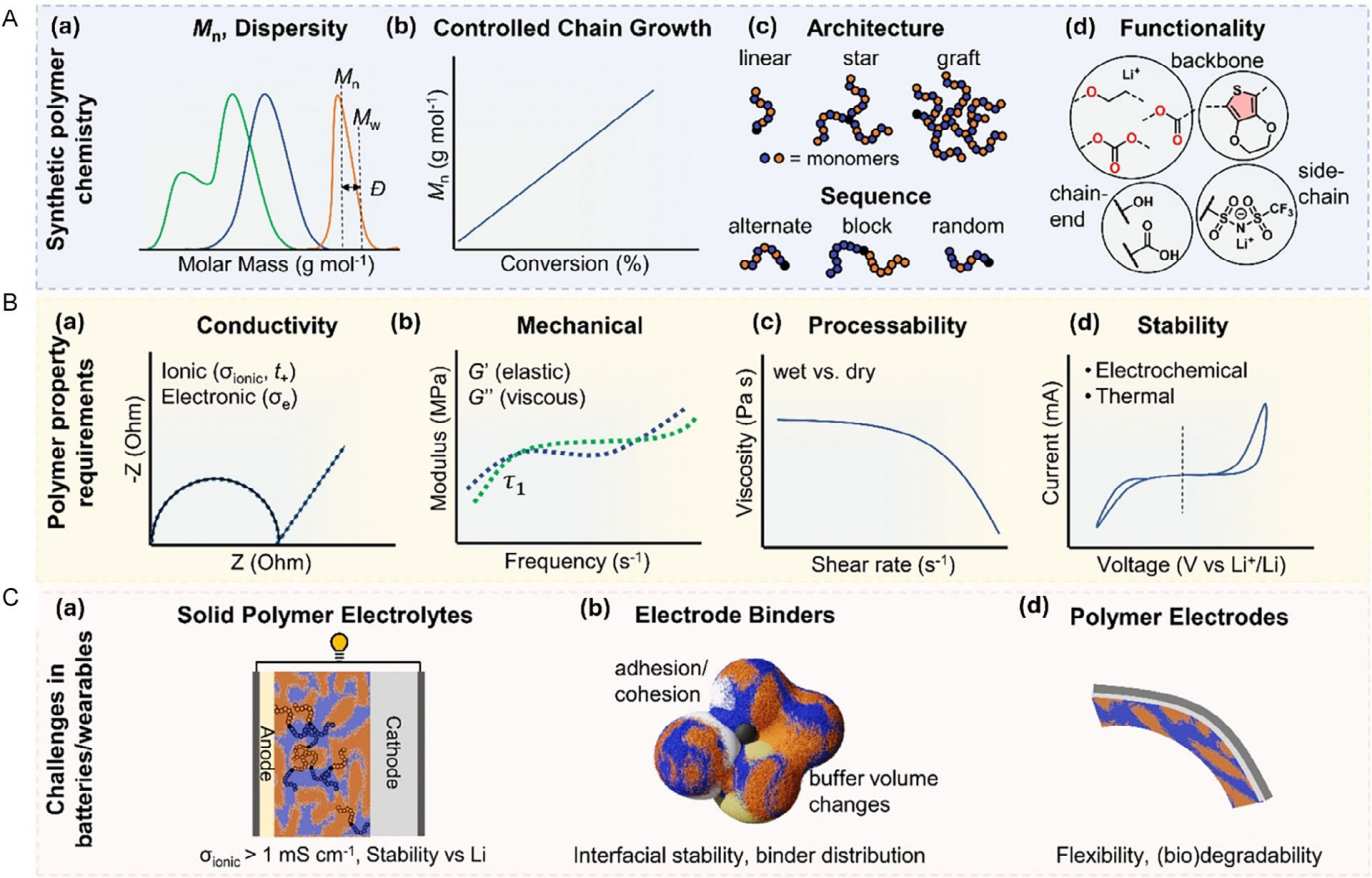
Schematic overview of polymer design strategies for SPE. (A) The synthetic polymer chemistry that enables (a) polymer molar mass (M_*n*_) and molar‐mass distribution (dispersity, Ð), (b) chain growth, (c) chain architecture, and (d) specific functional groups. (B) Key measurable polymer properties relevant to electrochemical energy storage, including (a) ionic conductivity, (b) mechanical, (c) processability, and (d) stability. (C) (a–c) Application‐driven requirements of SPEs in next‐generation solid‐state and flexible battery systems. Reproduced with permission from ref. [102]. Copyright 2024, The Royal Society of Chemistry.

SPEs have emerged as a strategically important class of electrolytes as battery research focuses on higher safety, higher energy density, and manufacturable solid‐state architectures [10, 15, 59, 98]. By replacing flammable liquid electrolytes with solvent‐free or quasisolid polymer media, SPE‐based cells can intrinsically reduce leakage and volatility risks while enabling thin, flexible membranes that are compatible with roll‐to‐roll processing, lamination, and scalable film manufacturing [99, 100, 101]. At the same time, polymer design offers a uniquely broad “chemical toolbox” (Figure 4)—including controlled polymerization, modular functional groups, and tunable network structures—allowing electrolyte properties to be engineered systematically rather than being dictated primarily by inorganic crystal chemistry [102]. Recent research efforts focus on innovative polymer design not only to improve ionic transport but also to deliver multifunctionality (adhesion, compliance, interlayers, and durability) across solid‐state and wearable battery platforms (Figure 4). Despite their appeal, SPEs remain constrained by two coupled limitations: (i) insufficient room‐temperature ionic conductivity in many fully solid polymer systems and (ii) interfacial resistance and mechanical degradation at solid–solid electrode contacts. The ion transport bottleneck is rooted in the fact that Li^+^ mobility in most classical SPEs is strongly tied to polymer segmental motion and local coordination–decoordination dynamics. Increasing conductivity often requires raising temperature, plasticizing the polymer, reducing crystallinity, or restructuring the polymer to create continuous polar domains—yet each lever can negatively affect modulus, oxidative stability, or interfacial robustness. Consequently, recent progress has increasingly come from SPE concepts that redistribute functions across structure and length scale: ultrathin membranes reduce Ohmic losses; scaffold reinforcement preserves dimensional stability; and network, topology, or block architectures are used to shape ion pathways without relying solely on bulk chain relaxation [103, 104, 105, 106].

**FIGURE 5:**
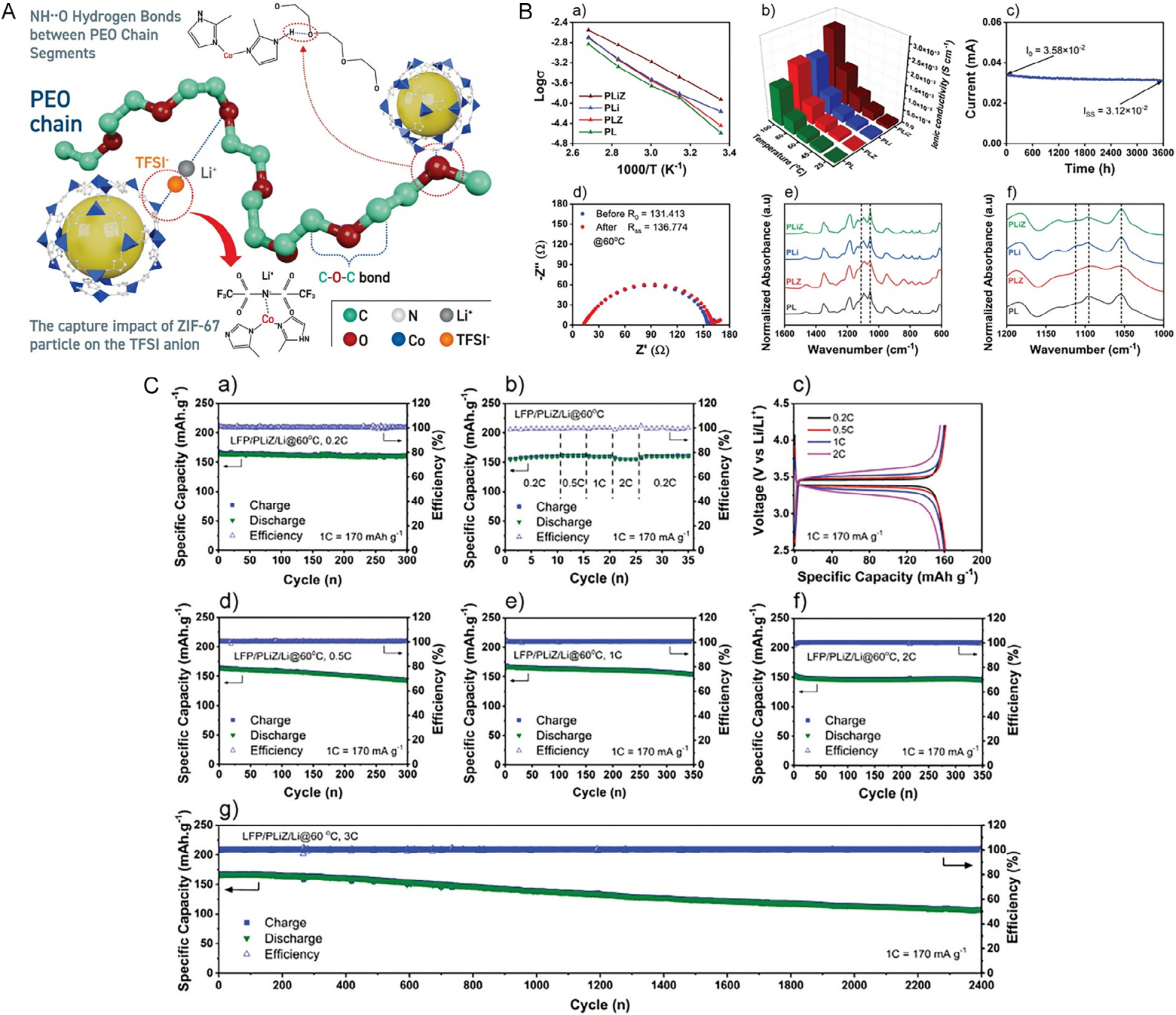
Solid‐state electrolyte based on modified PEO. (A) Schematic illustration of Li conduction in the MOF‐modified PEO chain. (B) (a) The ionic conductivity, (b) the ionic conductivities at different temperatures, (c) the chronoamperometry, (d) the Nyquist plot impedance of Li/PLiZ/Li symmetrical cells collected before and after polarization, (e) and (f) the FT‐IR spectra. (C) (a) The long‐cycle life evaluation of LFP/PLiZ/Li at current density of 0.2 C under 60 °C, (b) the rate capabilities, and (c) the charging/discharging curves of an integrated LFP/PLiZ/Li battery at different current densities of 0.2, 0.5, 1, and 2C at 60 °C; The long‐cycling performances of LFP/PLiZ/Li batteries at different current densities of (d) 0.5C, (e) 1C, (f) 2C, and (g) 3C at 60 °C. Reproduced with permission from ref. [107]. Copyright 2024, Wiley.

Recently, Nguyen et al. [107] reported a modified PEO/LiTFSI matrix with zeolitic imidazolate frameworks (ZIF‐67) and ionic liquid electrolytes as a potential SPE with improved properties. Through careful optimization of the fabrication process, ultra‐thin composite electrolyte membranes (denoted as PEO/LiTFSI–ILE–ZIF‐67, PLiZ) with a thickness of ≈32 µm were prepared. The resulting membranes exhibit a room‐temperature ionic conductivity of 1.19 × 10^−4^ S cm^−1^, a wide electrochemical stability window of up to 5.66 V, and a high lithium‐ion transference number of 0.8 (Figure 5). In parallel, substantial effort has been devoted to “beyond‐polyether” backbone chemistries, motivated by the oxidative instability and crystallization tendencies of PEO. Recent studies and reviews emphasize polymer matrices incorporating carbonyl groups (e.g., polycarbonates and polyesters), nitrile‐containing backbones, ionomers, and poly(ionic liquids), which offer alternative Li^+^ coordination environments and improved oxidative stability [20, 108]. These systems demonstrate that ionic conductivity and electrochemical window must be designed together at the backbone level as increased polarity alone does not guarantee fast ion transport if polymer dynamics or ion association are not simultaneously addressed. Beyond linear and functionalized polymer‐based SPEs, recent literature indicates a clear shift toward architecture‐driven SPEs, in which polymer topology and mesoscale structure are used to suppress crystallization and enhance the density and spatial organization of Li^+^ coordination sites. In this direction, “topological polymer electrolytes” (hyperbranched, star, comb, and brush) have gained attention as a pathway to improve dissociation and transport without relying on conventional plasticization strategies [109, 110, 111]. A representative study by Su et al. demonstrated the rational design of a topological polymeric solid electrolyte for high‐performance all‐solid‐state alkali‐metal batteries, illustrating how architectural control can shift the balance between conductivity, mechanical response, and interfacial behavior [112]. A subsequent review paper by Liu et al. consolidates these developments and clarifies why nonlinear architectures can outperform linear analogues through lower crystallinity and higher functional group density. Related “architecture‐first” approaches also include graft‐ and side‐chain‐engineered SPEs, where the backbone and side chains are codesigned to tune both segmental mobility and solvation environments; for instance, a report on solid‐state graft polymer electrolytes highlights the use of conductive backbones and side chains as a direct method to improve SPE performance [113]. Another important direction has been the development of ultrathin and scaffold‐supported SPEs, which address the conductivity limitation by reducing electrolyte thickness while preserving mechanical integrity. For example, PTFE‐reinforced polymer electrolytes with thicknesses on the order of tens of micrometers have demonstrated room‐temperature ionic conductivities approaching 10^−4^–10^−3^ S cm^−1^, wide electrochemical stability windows (>4.8 V vs. Li/Li^+^), and extended Li‖Li cycling lifetimes beyond 1000 h while remaining nonflammable and mechanically robust [114]. These designs highlight how structural reinforcement can decouple electrolyte thickness from mechanical failure, improving practical power density without compromising safety.

Collectively, these advances position modern SPEs as highly tunable material platforms rather than fixed host–salt systems (Table 1). While room‐temperature conductivity in fully SPEs still lags behind that of liquid electrolytes, the gap is narrowing through synergistic control of thickness, topology, and chemical functionality. Importantly, many of these strategies are compatible with existing polymer‐processing techniques, supporting their relevance for scale‐up and industrial implementation. The most promising SPE systems integrate multiple design concepts—architecture control, chemical tuning, and structural reinforcement—to balance conductivity, stability, and manufacturability.

**TABLE 1:** Performance metrics of SPE systems.

SPE system	**Conductivity σ, S cm** ^**−1**^	Li transference number, t_Li_^+^	Cyclic stability	Electrochemical window, V	Li‖Li stability	Reference
Scalable ultrathin SPE (high‐T tolerant)	—	—	300 cycles at 0.3 C rate at 60 °C	—	—	[101]
MOF‐assisted ultrathin SPE	1.19 × 10^−4^ S cm^−1^ at 25 °C	0.8	1000 cycles	5.66	—	[107]
PTFE‐reinforced ultrathin dual‐salt SPE	~ 4.5 × 10^−4^ S cm^−1^	—	>1000 cycles@2C	4.91	>1200 h	[114]
Polymerized ionic‐liquid SPE	~ 8 × 10^−4^ S cm^−1^	0.44	300 cycles	5.0	>1700 h	[115]
Flexible IL‐based SPE	1.24 × 10^−3^ S cm^−1^	0.47	100 cycles	4.8	—	[116]
Reinforced PEO‐based SPE	3.34 × 10^–5^ S cm^–1^ at 30 °C	0.68	160 cycles	5.0	1800 h	[117]
Topological polymeric SPE	6.43 × 10^−4^ S cm^−1^ at 80 °C	0.88	>200 cycles	6.0	—	[112]
Graft polymer SPE (architecture‐engineered)	~ 2 × 10^−5^ S cm^−1^at 30 °C	0.67	>100 cycles	4.5	—	[113]
Supramolecular self‐supporting SPE	1.1 × 10^−4^ S cm^−1^	0.53	—	—	700 h	[118]
Ultrathin polycarbonate SPE (≈7.8 µm)	4.8 × 10^−4^ S cm^−1^	0.68	1500 cycles	4.5	6000 h	[119]
Ultrathin polymer‐based solid electrolyte (12.4 µm)	~ 1 × 10^−3^ S cm^−1^	0.31	100 cycles	5.5	2100 h	[120]
In situ construction of an ultrathin SPE (≈5 μm)	2.0 × 10^−5^ S cm^−1^	—	500 cycles	≈4.28	800 h	[121]
In situ polymerized electrolyte enabling high‐voltage (4.6 V) full‐cell	6.93 × 10^−3^ S cm^−1^	0.84	300 cycles	6.0	—	[122]
Ultra‐thin and high‐voltage‐stable Bi‐phasic SPE	1.9 mS cm^−1^	0.56	250 cycles	4.9	—	[103]
Ultrathin multifunctional SPE (≈7 μm)	165 mS at 25 °C	—	1500 cycles	4.7	1600 h	[123]
Li^+^ affinity ultra‐thin SPE (35 µm)	4.26 × 10^−4^ S cm^−1^ at 50 °C	—	580 cycles	4.8	700 h	[106]
Nonflammable, ultra‐thin, high‐performance PEO‐based SPE	—	—	800 cycles	5.2	900 h	[124]
Ultrathin SPE (PVDF‐HFP)	1.21 × 10^−3^ S cm^−1^	—	200 cycles	4.85	9000 h	[125]

### 3.2 Self‐Healing Polymer Electrolytes

**FIGURE 6:**
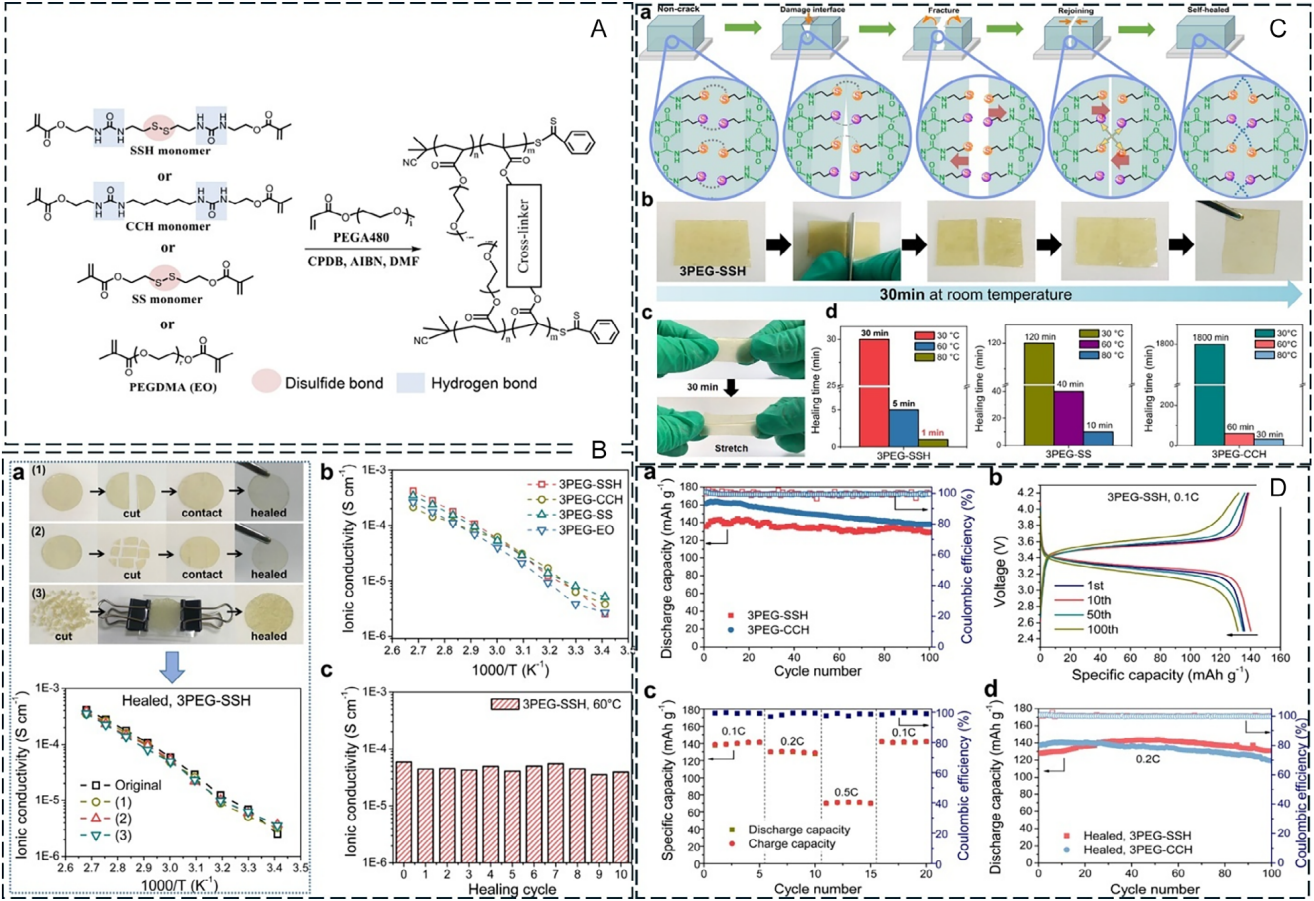
Self‐healing polymer electrolytes for next‐generation solid batteries [142]. (A) Schematic illustration of the polymerizations of PEGA and four types of cross‐linker. (B) (a) photographs of disk‐shaped 3PEG‐SSH cut into different forms. (Lower) temperature dependence of ionic conductivity of healed 3PEG‐SSH. (b) Temperature dependence of ionic conductivity of SPEs. (c) Ionic conductivity of healed 3PEG‐SSH after 10 cutting−healing cycles. (C) (a) Schematic illustration of the self‐healing process with disulfide bond and hydrogen bond. (b) 3PEG‐SSH film healed for 30 min at room temperature. (c) Stretching deformation of the healed 3PEG‐SSH film. (d) Self‐healing time of various SHSPEs. (D) (a) Cycling performances of Li|3PEG‐SSH|LFP and Li|3PEG‐CCH|LFP cells at a specific current of 0.1 C. (b) Charge−discharge curves with 3PEG‐SSH at different cycles at 0.1 C with the voltage range from 2.5 to 4.2 V. (c) Cycling of Li|3PEG‐SSH|LFP battery at 0.1, 0.2, and 0.5 C. (d) Cycling of Li|3PEG‐SSH|LFP and Li|3PEG‐CCH|LFP cells with healed SPEs at 0.2C [142]. Reproduced with permission from ref. [142]. Copyright 2020, The American Chemical Society.

Self‐healing polymer electrolytes are being developed to address a recurring practical weakness of SPEs: mechanical damage and interfacial degradation during cycling [126, 127]. Cracking of the electrolyte, loss of intimate electrode contact, and stress accumulation during Li plating/stripping can accelerate impedance growth and trigger localized current hotspots. Self‐healing concepts aim to restore ionic pathways and, in some designs, re‐establish conformal electrode–electrolyte contact without dismantling the cell [128, 129, 130]. Recent work has focused mainly on intrinsic self‐healing, achieved through reversible supramolecular interactions (e.g., hydrogen bonding and metal–ligand coordination) [131, 132] or dynamic covalent chemistry (e.g., imine exchange, disulfide exchange, and transamination) [133] because these mechanisms can enable repeated healing over many cycles rather than relying on finite, capsule‐based healing agents [134]. A widely used route is to introduce dynamic covalent bonds into the polymer network so that chain rearrangement and rebonding can occur after mechanical rupture [135, 136]. For example, imine‐bond chemistry has been used to construct self‐healing SPEs that maintain ionic transport while improving mechanical resilience and cycling stability in Li metal configurations [137]. Similarly, Liu et al. reported self‐healing SPEs, emphasizing the role of dynamic bonding in preserving electrolyte integrity and stabilizing metal interfaces during repeated cycling [138]. Another important direction is recyclable self‐healing network electrolytes, in which dynamic cross‐linking not only heals damage but also enables reshaping or recycling—an aspect increasingly aligned with sustainability‐driven electrolyte design. A representative example is a recyclable, self‐healing SPE derived from a bio‐based dynamic network (soy protein), which demonstrates how dynamic bonding can support both functional recovery and end‐of‐life handling concepts [139]. Self‐healing concepts have also been used to reinforce PEO‐based SSEs through tailored interfacial interactions; for instance, incorporation of amorphous 3D carbon into a PEO matrix has been reported to promote crack repair and sustain long‐term Li symmetric cycling and full‐cell operation by improving adhesion and mechanical robustness [140]. Solid electrolytes are engineered to heal not only bulk fractures but also electrode contact loss. An interfacial self‐healing polymer electrolyte incorporating dynamic disulfide bonds and hydrogen bonding was reported to reduce interfacial resistance and maintain stable contact in solid‐state Li‐metal cells, underscoring that “healing” can be targeted at the interface as much as the bulk [126]. Complementary dynamic‐chemistry approaches continue to emerge, including self‐curing/self‐healing SSEs based on bond‐exchange reactions (e.g., transamination), which can improve reliability during repeated cycling and reduce sensitivity to mechanical damage [141]. Overall, the recent literature over the past 5 years (2020–2025) indicates that self‐healing polymer electrolytes are moving from “proof‐of‐concept healing” toward battery‐relevant objectives: mitigating crack‐induced failure, slowing the growth of interfacial resistance, improving tolerance to deformation, and, in some cases, supporting recyclability. As a result, the most convincing recent strategies are those that treat self‐healing as one element of a broader electrolyte architecture—combined with interfacial engineering, controlled network structure, and conductivity‐enhancing chemistry—rather than as a stand‐alone feature (Figure 6) [142].

### 3.3 Block Copolymer Electrolytes

**FIGURE 7:**
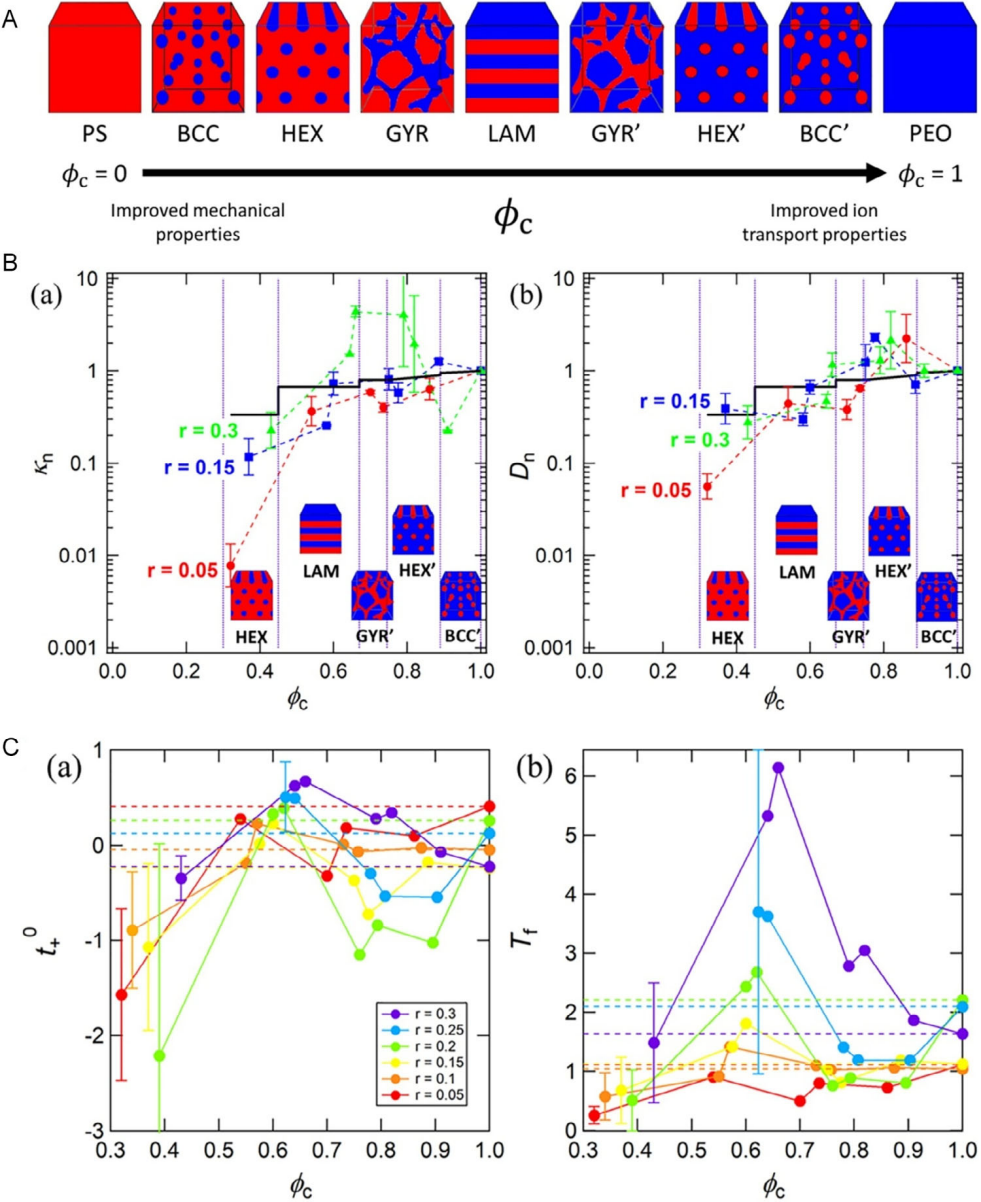
Polystyrene‐poly(ethylene oxide) (PS‐PEO) block copolymer electrolyte. (A) The PS‐PEO morphologies as a function of conducting phase volume fraction, *φ*_c_, with PS depicted in red and PEO with LiTFSI depicted in blue. As *φ*_c_ increases from *φ*_c_ = 0 (PS homopolymer) to *φ*_c_ = 1 (PEO homopolymer), the observed morphologies are BCC, body centered cubic spheres of PEO in a PS matrix, HEX, hexagonally packed cylinders, GYR, minority gyroid, LAM, alternating lamellae, GYR′, minority gyroid of PS in a matrix of PEO, HEX,′, hexagonally packed cylinders, BCC′, body centered cubic spheres. At low *φ*_c_, the electrolytes are rigid due to the continuous PS matrix but are poor ionic conductors. At high *φ*_c_, the electrolytes are highly conductive due to the continuous PEO matrix but not very rigid. (B) (a) Normalized conductivity, κ_*n*_, and (b) normalized salt diffusion coefficient, *D*_*n*_, as a function of conducting phase volume fraction, *φ*_c_, for *r* = 0.05 (red circles), *r* = 0.15 (blue squares), and *r* = 0.30 (green triangles). The vertical lines and illustrations indicate the volume fraction range where each morphology is observed. (C) (a) Cation transference number, t_+_^0^, and (b) thermodynamic factor, T_f_, of various PS‐PEO morphologies as a function of PEO volume fraction, *φ*_c_, at salt concentrations ranging from *r* = 0.05 to *r* = 0.30. Reproduced with permission from ref. [160]. Copyright 2020, The American Chemical Society.

Block copolymer electrolytes have emerged as an essential class of material platform for addressing the long‐standing trade‐off between ionic conductivity and mechanical stability in SPEs [143, 144, 145, 146, 147]. Unlike homopolymers, block copolymers consist of two or more chemically distinct polymer blocks that are covalently linked [148, 149]. Owing to the immiscibility of the blocks, these materials can undergo microphase separation, forming well‐defined nanostructures with characteristic length scales on the order of tens of nanometers [150, 151]. This intrinsic self‐assembly provides a means to spatially separate ion‐conducting and mechanically reinforcing domains within a single electrolyte [152]. In most block copolymer electrolyte designs, one block is selected for its ability to solvate lithium salts and support ion transport, while the second block provides mechanical rigidity. PEO [153, 154] is the most commonly used ion‐conducting block due to its strong coordination with lithium ions, whereas mechanically robust blocks such as polystyrene [155, 156], PMMA [157], or polycarbonate derivatives [26, 158] are often employed to enhance modulus and dimensional stability. Through appropriate block selection and composition control, it is possible to form continuous ion‐conducting pathways embedded within a mechanically stable framework. The morphology of block copolymer electrolytes plays a decisive role in their electrochemical performance [159]. Depending on the volume fraction of each block and the degree of segregation, ordered structures such as lamellae, cylinders, or gyroid phases can be obtained. Among these, morphologies that provide continuous pathways for the ion‐conducting block are particularly favorable for achieving high ionic conductivity (Figure 7) [160, 161, 162]. At the same time, the presence of rigid domains can suppress excessive polymer chain motion and improve resistance to lithium dendrite penetration, which is critical for lithium‐metal batteries.

**FIGURE 8:**
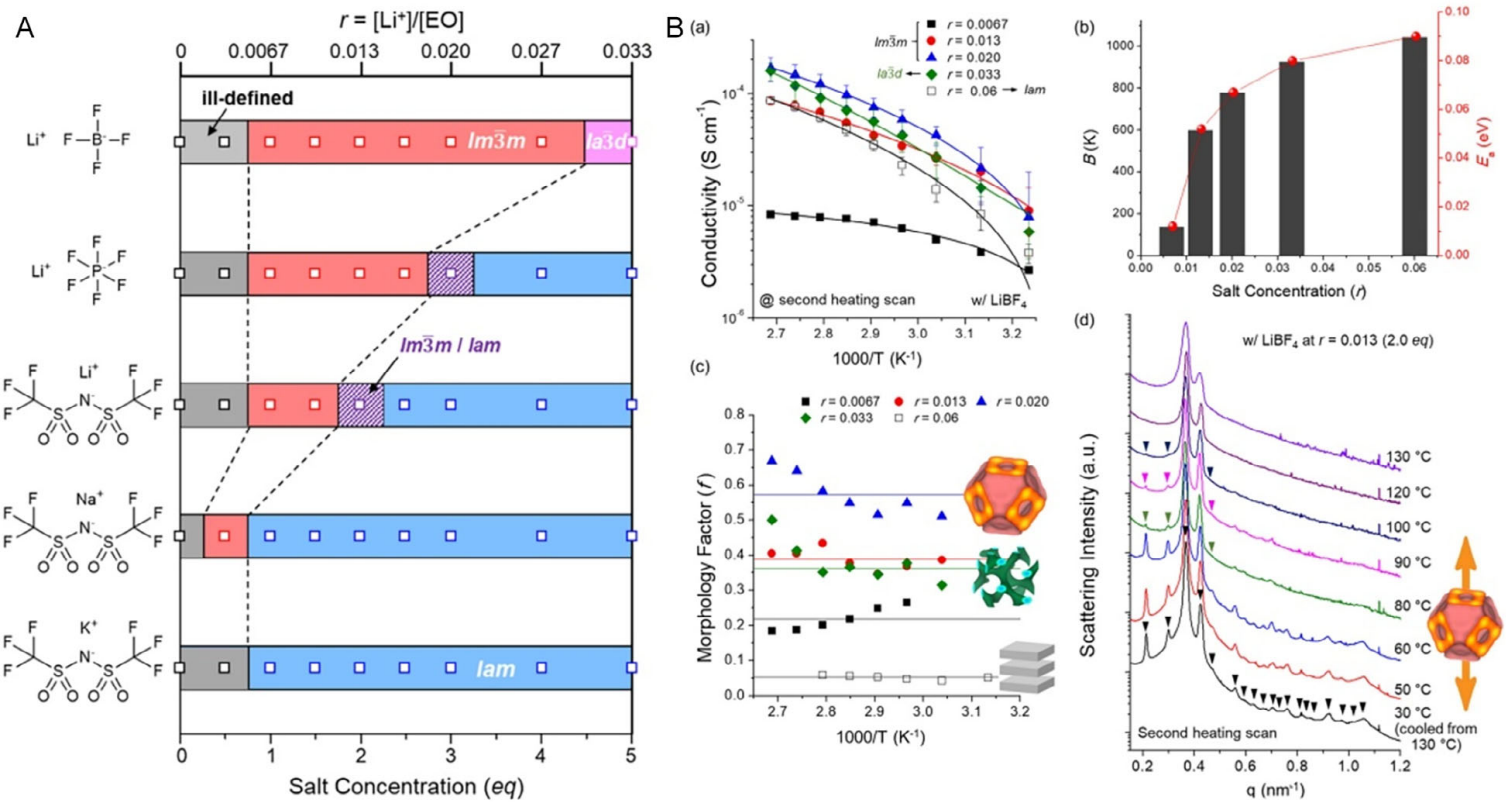
PS‐PEO block copolymer electrolyte with enhanced Li^+^ conduction through salt localization in double primitive cubic morphologies of the block copolymer [145]. (A) Phase diagrams of PS‐PEOs doped with different salts of different concentrations. (B) (a) Ionic conductivity of PS‐PEO electrolytes doped with LiBF_4_ as a function of temperature. (b) The energy required for ion motion (B) and activation barrier (*E*_a_). (c) Morphology factors. (d) SAXS profiles. Reproduced with permission from ref. [145]. Copyright 2025, The American Chemical Society.

One of the key advantages of block copolymer electrolytes is their ability to partially decouple ionic mobility from mechanical stiffness. In conventional polymer electrolytes, increasing mechanical strength often leads to reduced segmental motion and lower conductivity. In contrast, block copolymers allow mechanical reinforcement to be localized in specific domains, preserving ion transport in the conducting regions. A recent work by Lee et al. highlights enhancing lithium‐ion transport in block copolymer electrolytes by introducing trace amounts of lithium salts into (PS‐PEO) (Figure 8) [145]. At low salt concentrations, Li^+^ ions preferentially coordinate with the terminal ─OH groups of the PEO chains, promoting end‐to‐end interactions that drive the formation of a double primitive cubic (Im3̅m) morphology. Compared with TFSI^−^‐based salts, lithium salts containing smaller anions such as PF_6_^−^ and BF_4_^−^ more effectively localize Li^+^ ions, thereby broadening the composition window over which stable Im3̅m structures can be obtained. In PS‐PEO electrolytes doped with LiBF_4_ at lithium‐to‐ethylene oxide ratios of *r* = 0.013–0.02 (r ≡ [Li^+^]/[EO]), the resulting Im3̅m morphologies exhibit ionic conductivities several times higher than those observed in conventionally doped systems (e.g., *r* = 0.06). Analysis of the morphology factor reveals values more than eight times greater than those associated with lamellar‐forming electrolytes, indicating a highly efficient ion‐transport network. Consistent with this interpretation, the activation energy for lithium‐ion conduction in PS‐PEO containing one Li^+^ ion per PEO chain is as low as 0.012 eV when evaluated using the Vogel–Fulcher–Tammann model, suggesting that Li^+^ transport is only weakly coupled to polymer segmental relaxation. Further enhancement of the network structure can be achieved by chemically modifying the PEO chain ends with three ─PO_3_H_2_ groups, which strengthens Li^+^‐mediated end‐to‐end interactions and significantly extends the salt concentration range over which the Im3̅m morphology remains stable. By contrast, increasing the number of terminal ─OH groups via diol‐ or triol‐terminated PEO has little effect on stabilizing the 3D network, underscoring the importance of specific end‐group chemistry in controlling block copolymer electrolyte morphology and ion‐transport behavior.

Despite their promise, block copolymer electrolytes face several limitations. Their synthesis and processing are generally more complex than those of homopolymer electrolytes, and achieving well‐ordered morphologies often requires careful control of molecular weight, composition, and thermal history. In addition, many block copolymer systems still rely on PEO as the conducting block and thus inherit its intrinsic limitations at ambient temperatures. Efforts to incorporate alternative ion‐conducting blocks or to combine block copolymers with ceramic fillers and plasticizers are ongoing. Overall, block copolymer electrolytes represent a rational materials design strategy for solid‐state batteries, offering a unique route to engineer electrolyte structure at the nanoscale. Continued advances in polymer synthesis, morphology control, and ion‐conducting block development are expected to further improve their performance and clarify their role in practical SSB systems.

### 3.4 Single‐Ion Conducting Polymer Electrolytes (SIPEs)

**FIGURE 9:**
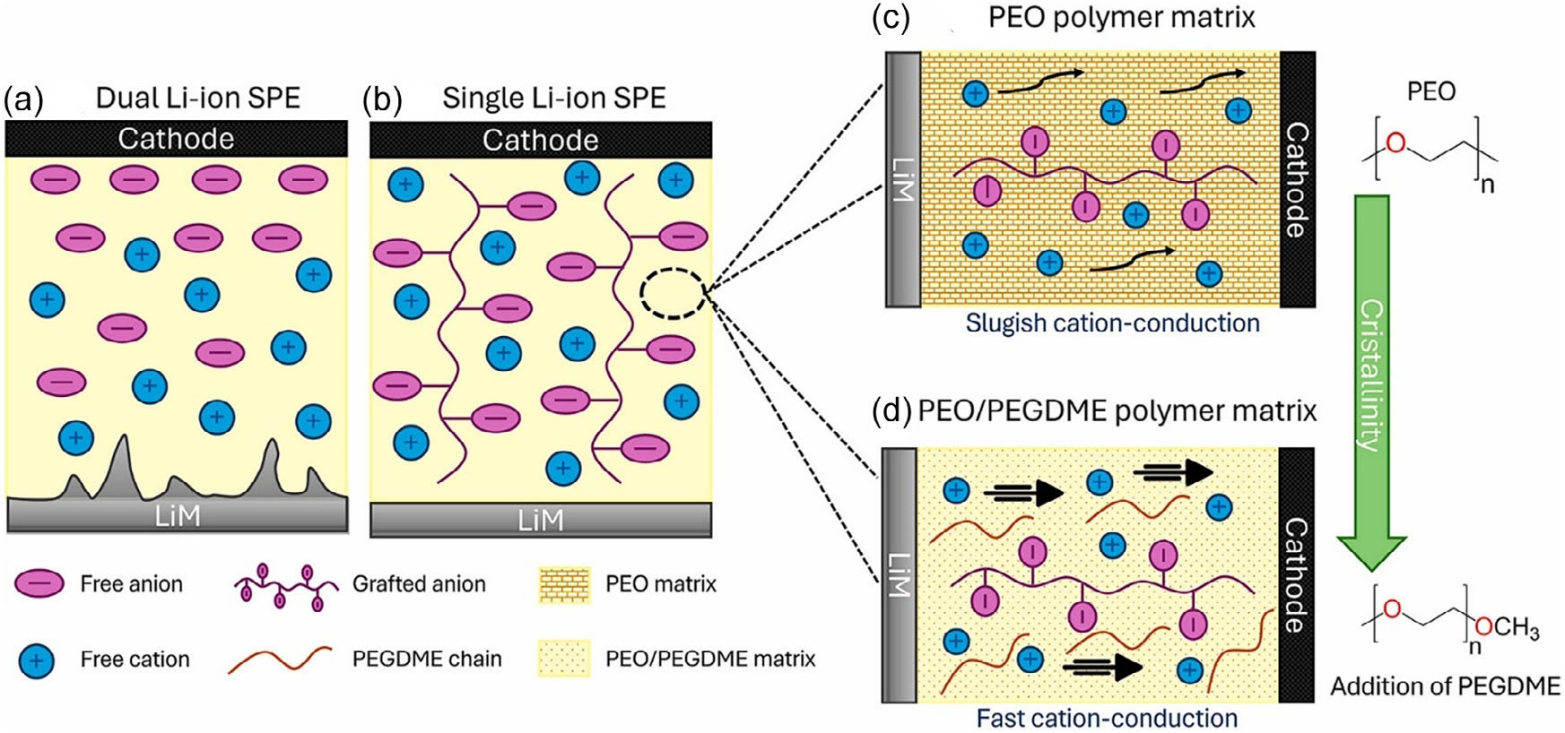
Li^+^ ion transportation in single‐ion polymer electrolytes (PEO/PEGDME) in comparison to that of conventional solid polymer electrolytes (PEO). (a) Dual Li‐ion SPE containing mobile cations and anions and (b) single Li‐ion SPE with immobilized/grafted anions and mobile Li^+^. (c) In the crystalline PEO matrix, restricted polymer‐chain mobility results in sluggish cation transport. (d) Addition of PEGDME reduces PEO crystallinity and increases chain mobility, producing a more favorable Li^+^‐transport environment and faster cation conduction. Reproduced with permission from ref. [163]. Copyright 2024, Elsevier.

SIPEs represent a distinctive class of SPEs in which the anionic species is tethered to the polymer backbone or immobilized, leaving only the lithium cation mobile (Figure 9) [163]. This architecture can deliver near‐unity Li^+^ transference numbers and substantially reduce concentration polarization under current flow, in contrast to dual‐ion‐conducting systems in which both cations and anions migrate. Design strategies for SIPEs aim to balance ion transport, mechanical integrity, and interfacial stability. A key rationale for pursuing SIPEs is their potential to suppress lithium dendrite formation via reduced concentration polarization. In dual‐ion systems, mobile anions can accumulate or deplete at electrode interfaces under current, leading to steep lithium concentration gradients that exacerbate nonuniform deposition and dendrite initiation. In contrast, immobilized anions in SIPEs prevent significant anion migration, thereby minimizing concentration polarization and reducing the driving force for dendritic growth, particularly at high current densities.

**FIGURE 10:**
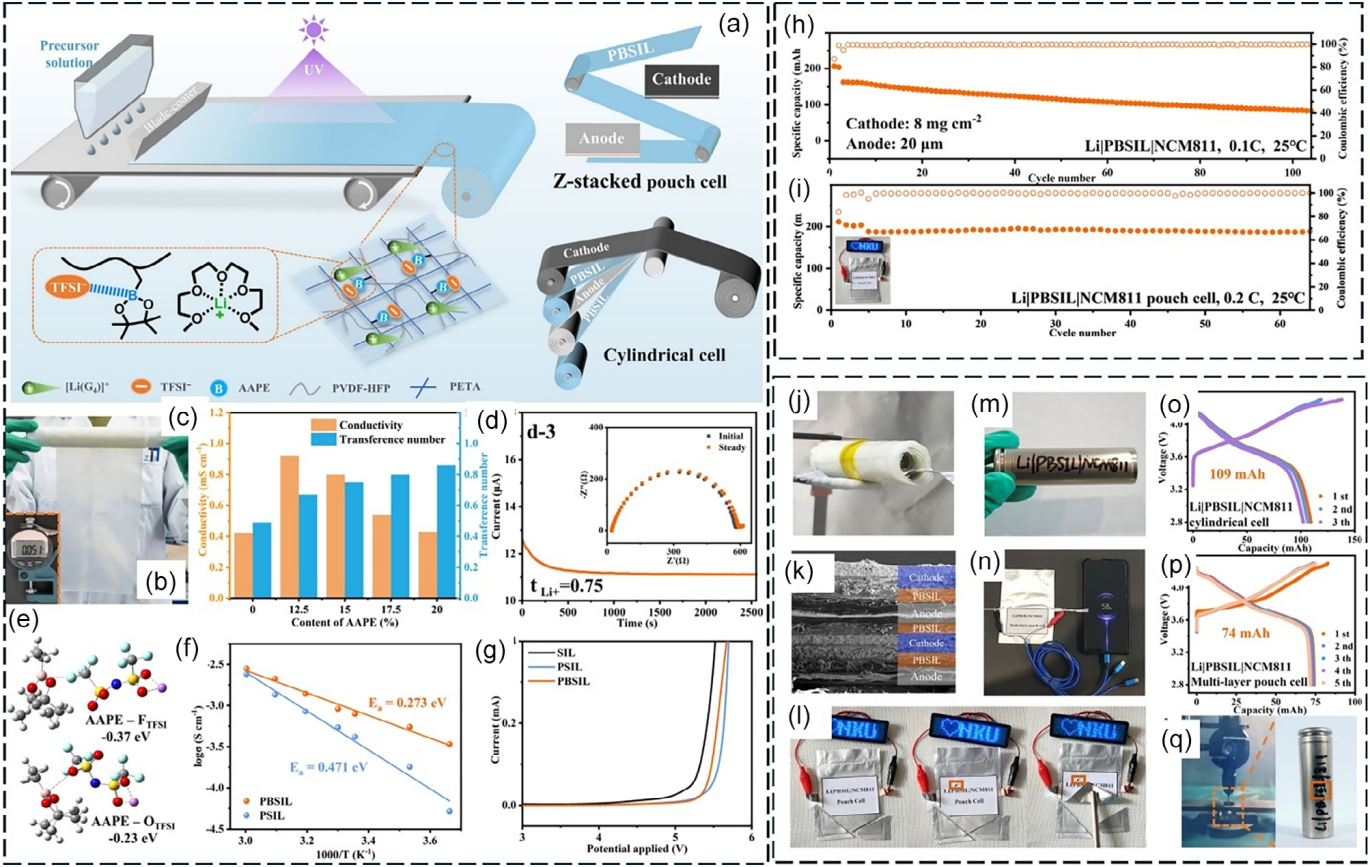
Preparation, scale‐up, and performance evaluation of a single‐ion polymer electrolyte system. (a) Schematic illustration of SIPE based on PVDF‐HFP (PBSIL), (b) optical image of the large‐scale SIPE, (c) ionic conductivities and Li^+^ transference numbers of the SIPEs with different wt% of anion acceptor (Borate ester, AAPE), (d) Li^+^ transference number, (e) adsorption energy between TFSI and AAPE. (f) Arrhenius plots different temperatures. (g) Linear voltammetry curves, (h) cycling performances of LiNCM811 cells prepared with PBSIL and the control PSIL and SIL systems at 0.2C, (i) long‐cycling performance of Li|PBSIL|NCM811 cell with charging at 0.2C and discharging at 0.5C. (j) and (k) Optical images of cylindrical cells (l) charge–discharge profiles of the cylindrical cell. (m) Cross‐sectional SEM image of Li|PBSIL|NCM811 multilayer pouch cell, (n) optical image of the pouch cell charging a phone, (o) charge–discharge profiles of the pouch cell, (p) optical images of pouch cell powering a light‐emitting diode screen under harsh conditions including bending, cutting and punching, and (q) optical images of cylindrical cell during the nail penetration test. Reproduced with permission from ref. [165]. Copyright 2024, The Royal Society of Chemistry.

Recent works emphasize tailored polymer backbones and tethered anion chemistries that weaken Li^+^–anion binding while preserving polymer segmental motion. For example, amorphous single‐ion conducting polymers with engineered perfluorosulfonate lithium units have shown improved ionic conductivity (>10^−5^ S cm^−1^ at 60 °C) and high transference numbers approaching unity, coupled with extended symmetric Li stripping/plating stability without evidence of dendritic shorting in bench cells [164]. Notably, Zhang et al. reported a SIPE demonstrating high ionic conductivity (~8.0 × 10^−4^ S cm^−1^) and a Li^+^ transference number of ~0.75, which was shown to mitigate concentration polarization and suppress dendrite growth in long‐cycle lithium cells [165]. free‐standing single‐ion conducting semi‐SPE (PBSIL) is developed by exploiting the cooperative interaction between anion‐accepting moieties and solvated ionic liquids. This design promotes efficient lithium salt dissociation while simultaneously immobilizing anionic species, thereby enabling preferential and rapid Li^+^ transport. When evaluated in Li|PBSIL|LiNi_0.__8_Co_0.__1_Mn_0.__1_O_2_ cells, the electrolyte delivers a discharge capacity of 183 mA h g^−1^ and maintains 75% capacity retention over 1300 cycles, demonstrating good electrochemical durability. Importantly, the PBSIL electrolyte is also compatible with practical cell manufacturing processes and has been successfully implemented in winding‐processed semisolid cylindrical cells as well as Z‐stacked pouch lithium‐metal batteries (Figure 10). These results indicate that regulating Li^+^ transport through anion immobilization provides an effective strategy for designing free‐standing polymer electrolytes with strong electrochemical performance, offering a viable pathway toward long‐cycling lithium‐metal batteries suitable for industrial application. Table 1 comprehensively summarizes representative SIPEs along with their performance metrics, including their conductivities, Li^+^ transference number, voltage window, and stability.

SIPEs offer a compelling transport regime for next‐generation solid‐state batteries, with recent studies demonstrating that optimized polymer backbone design, tethered anion chemistry, and structural hybridization can collectively improve practical performance (Table 2). Continued efforts to enhance ambient‐temperature conductivity, interface engineering with electrodes, and manufacturing scalability will be crucial in translating the inherent advantages of single‐ion conduction into commercial SSB systems. Despite such progress, room‐temperature conductivity remains a bottleneck for many SIPE systems, as polymer segmental mobility and tethered anion design must be co‐optimized to facilitate fast Li^+^ transport without sacrificing rigidity or interface compatibility.

**TABLE 2:** Performance metrics of representative SIPE systems.

SIPE system	σ, S cm^−1^	t_Li_^+^	Electrochemical window (V)	Li‖Li stability	Reference
Free‐standing SIPE film	10^−5^ at 60 °C	0.9	4.0	80 days	[164]
free‐standing single‐ion conducting semisolid polymer electrolyte	Up to 9.2 10^−4^	0.75	5.1	3000 h	[165]
Elastic SIPE networks	~10^−6^–10^−5^	—	4.0	—	[166]
Sulfonate‐tethered SIPE	~10^−6^	0.84	5.53	1000 h	[167]
Borate‐pendant SIPE	1.65 × 10^−4^ at 60 °C	0.93	4.2	175 h	[168]
Photo‐cross‐linked SIPE	0.21 mS cm^−1^ at 20 °C and 0.40 mS cm^−1^ at 40 °C	—	4.0	1000 h	[169]
Sulfonylimide‐based SIPE	7.8 × 10^−5^ (30 °C)	0.95	4.4	—	[170]
Tough SIPE membrane	∼ 7 × 10^−6^ at 70 °C	0.9	—	>1200 h	[171]
Aza‐linked SIPE	2.7 × 10^–4^ at 30 °C; 1.4 × 10^−3^ at 80 °C	—	—	600 h	[172]
SIPE–garnet hybrid	8.6 × 10^−5^ at 30 °C	0.73	~5.0	700 h	[173]
Polymer blend SIPE	7 × 10^−3^ at 150 °C	0.9	4.7	—	[174]
Fluorine‐free SIPE	>10−4 S at 40 °C	>0.9	4.1	1000 h	[175]
Hydrocarbon‐based SIPE	5.4 × 10^−5^ at 30 °C	—	5.0	>400 h	[176]
Lithiated Nafion membrane as a SIPE	5.5 × 10^–4^ at 60 °C	—	3.0	130 h	[177]
MOF‐modified SIPE	4.57 × 10^–6^ at 25 °C	≥0.8	—	2000h	[178]

### 3.5 Polymer–Ceramic Composite Electrolytes

Polymer–ceramic composite solid electrolytes were widely studied, as they combine the high ionic conductivity of ceramics with the mechanical flexibility and processability of polymer matrices (Table 3). By integrating these two distinct phases, composite electrolytes aim to overcome the intrinsic limitations of single‐phase polymer or ceramic systems, particularly the low room‐temperature conductivity of polymers and the brittleness and poor interfacial contact associated with ceramics. In most composite designs, ceramic fillers are dispersed within a continuous polymer matrix. Depending on their function, these fillers can be broadly classified as either inert or active. Inert fillers, such as Al_2_O_3_ or SiO_2_, do not conduct lithium ions themselves but can enhance ionic conductivity indirectly by suppressing polymer crystallization, increasing segmental mobility, or promoting lithium salt dissociation through Lewis acid–base interactions. In contrast, active ceramic fillers, including garnet‐type, NASICON‐type, or sulfide‐based lithium‐ion conductors, can directly participate in ion transport and potentially provide fast conduction pathways within the composite.

**TABLE 3:** Summary of polymer–ceramic composite solid electrolytes.

Sample name	Polymer matrix	Filler, motif	σ, S/cm	Modulus, toughness	Potential window, V	Processing, morphology	References
PEO + Al_2_O_3_	PEO	2D γ‐Al_2_O_3_ nanosheets	2.02 × 10^−4^ @ 25 °C	↑ versus neat PEO; flexible films	4.8	Solution cast	[63, 179, 180]
PEO + TiO_2_	PEO	TiO_2_ (5–8 nm)	2.9 × 10^–4^ @ 70 °C	Improved modulus versus neat PEO	—	Solution cast	[63, 179, 181]
PEO + SiO_2_ (fumed)	PEO	SiO_2_	2.9 × 10^–4^ @ 25 °C	Toughness ↑; crystallinity ↓	4.0	Solution cast	[52, 182]
PEO + SiO_2_	PEO	Hollow mesoporous SiO_2_	1.07 × 10^−4^ @ 30 °C	—	4.73	Solution cast	[183]
PEO + Succinonitrile (SN)and ZnO nanoparticles	PEO	SN and ZnO nanoparticles	1.1 × 10^−4^ @ RT	—	—	Solution cast	[184]
PEO + LLZO nano	PEO	LLZO (garnet) nanoparticles	1.1 × 10^–7^ ± 2.0 × 10^–8^	Reinforced	—	Hot‐press	[185]
PEO + LLZO	PEO	Nano LLZO	1.1926 × 10^–4^ @ RT	Reinforced	5.5	Solution cast	[186]
PVDF‐HFP Al‐doped LLZO nanofibers	PVDF‐HFP	Al‐doped LLZO	3.26 × 10^−4^	High path continuity; strength ↑	—	Electrospun scaffold	[187]
PEO + LATP nano	PEO	LATP nano	1.2 × 10^−4^ S cm^−1^ @ 60 °C	Modulus ↑; interface care at anode	4.9	Solution cast	[188, 189]
PEO + LATP nanofibers	PEO	LATP nanofibers (1D)	3.82 × 10^−4^	Percolated network → σ↑	4.8	Electrospun	[190, 191]
PEO + LAGP nano	PEO	LAGP nano	4.63 × 10^−4^	Phosphate chemistry	5.1	Solution cast	[192]
PVDF‐LLZO	PVDF	LLZO	2.43 × 10^–4^ @ RT	Interphase‐forming, improved adhesion	—	Solution cast	[81]
3D‐MOF‐Cellulose/SN/PEO	Cellulose‐SN/PEO	ZIF‐67	1.17 × 10^–4^ @ 30 °C	Robust membrane	5.0	Solution cast	[193]
polyethylene glycol‐perfluoropolyether (PEG‐PFPE)‐Al_2_O_3_	PEG‐PFPE	Al_2_O_3_	1.21 × 10^−4^ @ RT	High path continuity	5.0	In situ polymerization	[194]
PI–PEO_0.2_–PDA@LATP_0.8_	PI‐PEO	PDA@LATP	2.07 × 10^−4^ at 30 °C	Tough, good film handling	5.2	Electrospun	[195]
3D LLZO‐PAN	PAN	LLZO nano	2.9 × 10^−4^	3D‐conductive self‐supporting network	4.9	Electrospun	[196]
In situ polymer‐coated LLZTO	PMMA, PEO, PVP, and PS	LLZTO	LLZTO@PMMA, LLZTO@PEO, and LLZTO@PVP electrolytes exhibited high ionic conductivities of 3.00 × 10^−5^ S cm^−1^, 2.91 × 10^−5^ S cm^−1^, and 2.67 × 10^−5^ S cm^−1^ at 25 °C, respectively.	—	—	Wet Ball‐milling	[197]
Interpenetrating network polycarbonate‐LLZO	Polycarbonate (PC)	LLZO	3.56 × 10^−4^	Enhanced mechanical properties, including a high tensile strength to isolate the electrode and improve the safety of the battery, and a high Young's modulus to suppress the formation of lithium dendrite	5.0	In situ polymerization	[198]
Poly(ethylene carbonate)‐Al_2_O_3_	Polycarbonate	Al_2_O_3_	2.19 × 10^−5^	—	4.6	Solution cast	[199]
PVDF/LiTFSI/Al‐LLZO	PVDF	Al‐LLZO	1.712 × 10^−4^ @ 25 °C	—	4.9	Electrospun	[200]
SiO_2_ functionalized PEGDE and PEA‐PVDF‐HFP	Polyethylene glycol diglycidyl ether (PEGDE) and polyetheramine (PEA) as cross‐linking point	SiO_2_	2.4 × 10^−4^	Tough films; σ↑ at RT	—	Solution cast	[201]
PEO/PAN/SiO_2_	PEO/PAN	SiO_2_	2.03 × 10^−7^	—	5.0	Solution cast	[202]
Ti_3_C_2_T_*x*_ MXene enhanced PEO/SN	PEO/SN	MXene	2.17 × 10^−3^ @ 35 °C	The 2D nanofillers provide a rigid confinement network that inhibits PEO crystallization when a moderate amount of Ti_3_C_2_T_*x*_ is added.	—	Solution cast	[203]
MXene‐PEO‐LFP	PEO	MXene (Ti_3_AlC_2_)	1.1 × 10^−3^	—	—	Solution cast	[204]
PEO‐Ti_3_C_2_T_*x*_	PEO	Ti_3_C_2_T_*x*_	5.01 × 10^−2^ @ 60 °C	Excellent flexibility	5.1	Tap casting and hot pressing.	[205]
PEO/Nb_2_CT_*x*_ MXene	PEO	Nb_2_CT_*x*_ MXene	2.62 × 10^−4^ @ 60 °C	Reinforced	5.0	Solution cast	[206]
Ti_3_C_2_T_*x*_ MXene/PAN nanofiber and LLZTO/PEO	PEO‐PAN	LLZTO/MXene	2.17 × 10^−4^ @ 30 °C	Reinforced mechanical properties	5.2	Electrospinning	[207]
Injection‐molded PEO/MXene	PEO	Ti_3_C_2_T_*x*_	1.2 × 10^−4^	Improved mechanical properties	—	Injection molding	[208]
Chemically Couple‐Linked MXene‐PEO	PEO	Ti_3_C_2_T_*x*_	5.6 × 10^–4^	—	4.7	Solution cast	[209]
Ionic liquid‐grafted MXene composite polymer	PEO	Ti_3_AlC_2_	7.19 × 10^−4^	—	5.2	Solution cast	[210]
MXenes methoxypolyethylene glycol (mPEG) modified MXenes/poly(vinylidene fluoride)‐hexafluoropropylene matrices, enhanced with succinonitrile as a plasticizer	PVDF‐HFP	Ti_3_AlC_2_	1.49 × 10^−4^ @ 30 °C	Improved ductility	4.75	Solution cast	[211]
MXene‐coordinated polymer electrolyte (MCPE)	Triethylene glycol dimethacrylate (TEGDMA) and polyethylene glycol monomethyl ether methacrylate (PEGMEMA)	Ti_3_AlC_2_	7.685 × 10^−4^ @ 25 °C	Higher energy storage modulus	5.03	In situ polymerization	[212]
(2D) fluorinated graphene reinforced PVDF‐HFP‐LiTFSI	PVDF‐HFP‐	Fluorinated graphene	1.32 × 10^–4^ @ 30 °C	Higher breaking strength and total elongation	—	Doctor blading	[213]
Sandwich structured metal oxide/reduced graphene oxide(rGO)/metal oxide nanocomposites (PEO‐ZnO/rGO/ZnO)	PEO	ZnO/rGO	1.02 × 10^−4^ @ 25 °C	High ultimate tensile stress of 2.09 MPa and Young's modulus of 2.71 MPa	5.3	Solution cast	[214]

**FIGURE 11:**
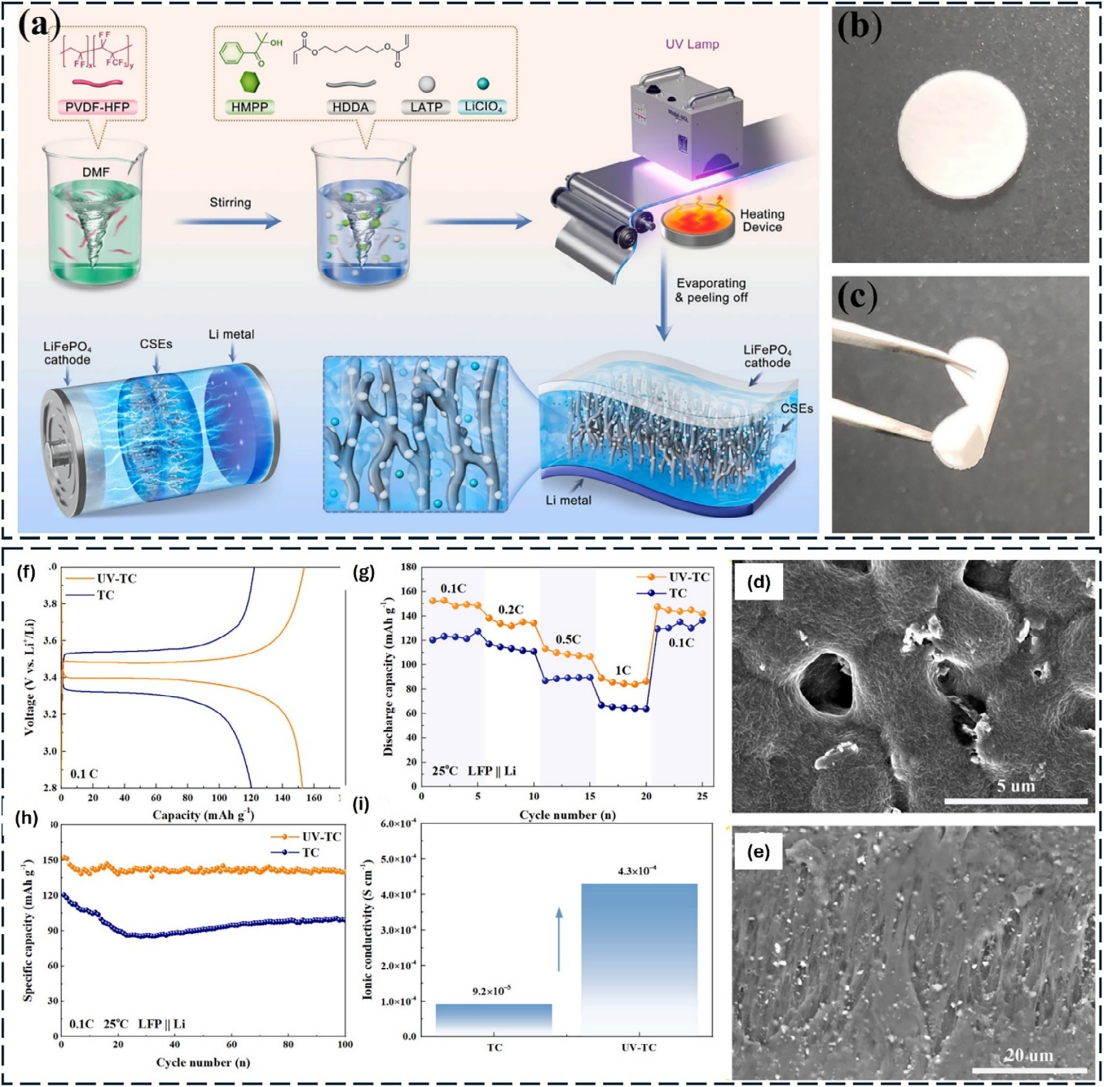
Preparation and performance evaluation of polymer–ceramic composite electrolyte. (a) Schematic representation of the preparation of PVDF‐HFP‐LATP‐LiClO_4_ composite electrolyte using thermo‐couple heating (TC) and UV‐combined TC (UV‐TC). (b,c) Photographs of the composite electrolyte, actual and bent, respectively. (d) FE‐SEM. (e) Top‐view. (f) Cross‐sectional view of the electrolyte. (g) Charge–discharge curves. (h) Rate performance. (i) Cycling performance at 0.1C. Ionic conductivity. Reproduced with permission from ref. [215]. Copyright 2024, Elsevier.

The performance of polymer–ceramic composite electrolytes is strongly influenced by their preparation method, which governs filler dispersion, interfacial contact, and overall microstructure. Among the most commonly employed approaches, solution casting is widely used at the laboratory scale. In this method, the polymer, lithium salt, and ceramic fillers are dispersed in a suitable solvent to form a homogeneous slurry, which is then cast and dried to obtain free‐standing electrolyte films (Figure 11) [215]. Solution casting enables good control over composition and film thickness, but the use of organic solvents and sensitivity to moisture can complicate scale‐up and reproducibility. Melt processing and hot pressing offer solvent‐free alternatives that are more compatible with industrial manufacturing. These techniques rely on heating the polymer above its melting temperature to enable uniform mixing with ceramic fillers, followed by compression into dense electrolyte membranes. Melt‐based methods can improve interfacial contact and reduce residual solvent effects, although high processing temperatures may limit the choice of polymer and ceramic components. In situ polymerization has also been explored as an effective route to improve ceramic dispersion and interfacial bonding. In this approach, monomers or oligomers are polymerized directly in the presence of ceramic fillers, often within porous electrodes or preformed scaffolds. This method can generate intimate ceramic–polymer interfaces and reduce particle aggregation but requires careful control of polymerization kinetics and reaction compatibility.

**FIGURE 12:**
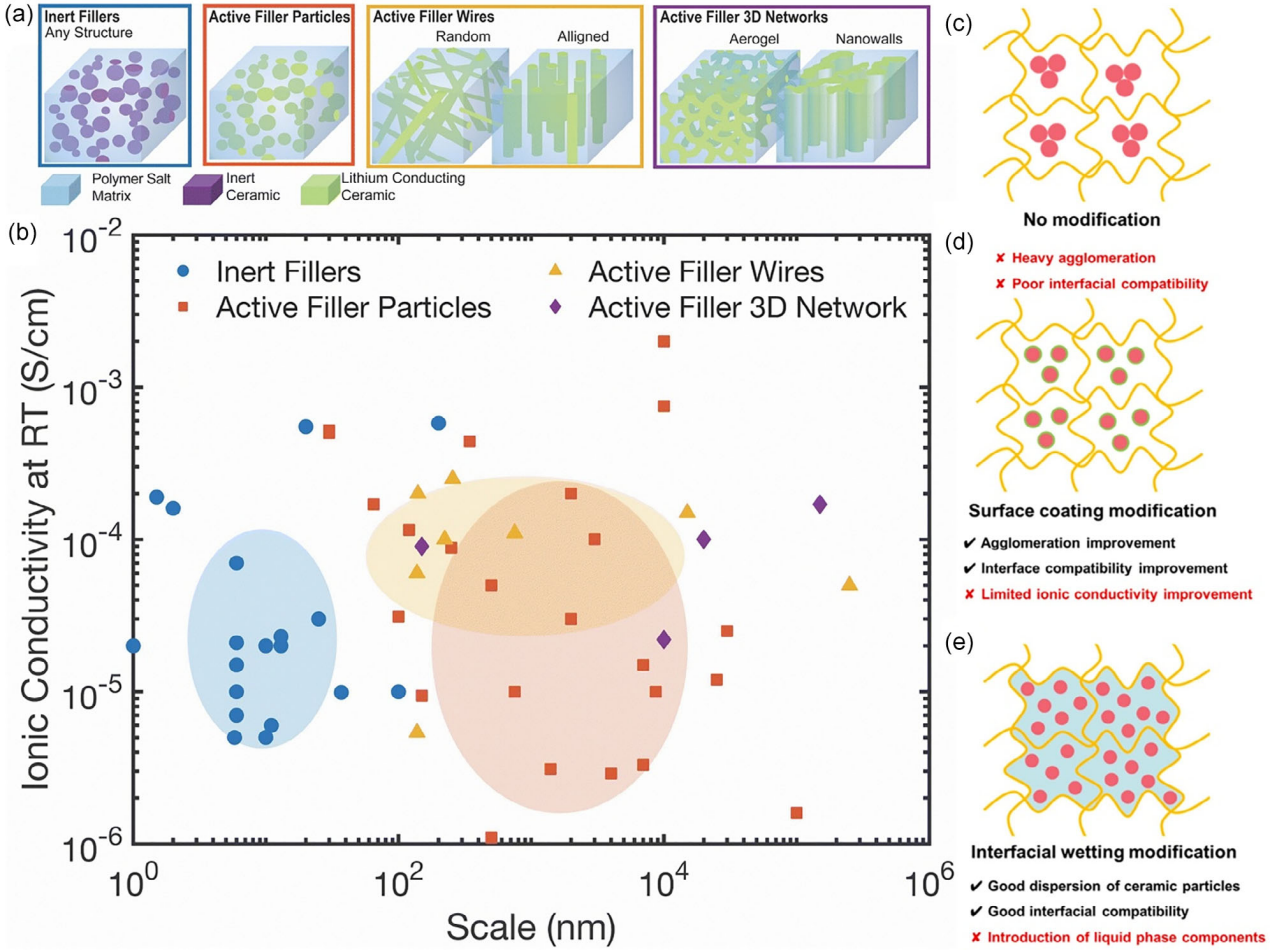
Effect of dimension, morphology, and Li^+^ conductivity of ceramic materials on the ionic conductivity of polymer–ceramic composite electrolytes (Li^+^ conducting ceramics are represented as active fillers while Li^+^ nonconducting ceramics are defined as inert fillers). (a) Graphic representations of inert and active filler particles in polymer matrix and active ceramic matrices of different morphologies, Reproduced with permission from ref. [216]. Copyright 2025, Elsevier. (b) Summary of their ionic conductivities. (c) Common challenges associated with incorporating unmodified ceramic particles with polymer matrices. (d) Property enhancement with surface modification of fillers and (e) further performance enhancement with interfacial wetting modifications. Reproduced with permission from ref. [33]. Copyright 2025, The Royal Society of Chemistry.

While such composites are often designed with the expectation that the ceramic phase provides rapid ion‐transport pathways, experimental studies have reported ionic conductivities that exceed these expectations [33]. In several cases, significant conductivity enhancements have been reported even when the ceramic content remains below the percolation threshold or when the incorporated ceramic fillers are not themselves lithium‐ion conductors (note that the fillers that are nonionic conductive are inert fillers, whereas the ones that are ionic conductive are active fillers) (Figure 12a,b) [33, 217, 218, 219, 220]. For instance, Sivaraj et al. reported a composite electrolyte PVDF/LiClO_4_/Li_0.5_La_0.5_TiO_3_ containing 30 wt% of Li_0.5_La_0.5_TiO_3_ (LLTO), corresponding to ≈20 vol%, dispersed in a PVDF polymer matrix [220]. If one assumes that each phase in the composite contributes independently to its respective bulk ionic conductivity, the expected lithium‐ion conductivity can be estimated using a simple volume‐weighted average. Based on the conductivity values reported for the individual components, this approach yields a predicted conductivity of ≈1 × 10^−4^ S cm^−1^. In contrast, the experimentally measured conductivity of the composite was substantially higher, reaching 2.7 × 10^−3^ S cm^−1^ [220], more than an order of magnitude higher than the value anticipated from bulk‐phase contributions alone. This nonlinear dependence of Li^+^ conductivity strongly suggests that mechanisms beyond direct ion transport through the ceramic phase may contribute to the overall conductivity of polymer–ceramic composites. Possible explanations include modified polymer chain dynamics, enhanced salt dissociation at polymer–ceramic interfaces, or the formation of interfacial conduction pathways. However, the relative importance of these effects remains unclear, and a unified understanding has yet to emerge. Further systematic investigation is therefore required to clarify the origins of the unusually high ionic conductivity observed in these composite electrolytes. The morphology and dispersion of the ceramic phase play a critical role in determining composite performance. Agglomeration of ceramic particles can disrupt continuous ion‐conduction pathways and degrade mechanical properties, whereas uniform dispersion promotes effective use of interfacial regions. As a result, surface modification of ceramic fillers, particle size control, and optimized processing routes have become important aspects of composite electrolyte design. In addition, the mechanical properties of polymer–ceramic composites can be tuned by adjusting filler content and polymer chemistry, enabling improvements in modulus and resistance to lithium dendrite penetration. Despite their advantages, polymer–ceramic composite electrolytes also present challenges. High ceramic loadings, while beneficial for conductivity and mechanical reinforcement, can compromise flexibility and processability. Chemical incompatibility between certain polymers and ceramic electrolytes, particularly sulfide‐based materials, may lead to interfacial degradation or undesirable side reactions. Furthermore, the complexity of ion‐transport mechanisms in these heterogeneous systems complicates rational design and performance optimization.

**FIGURE 13:**
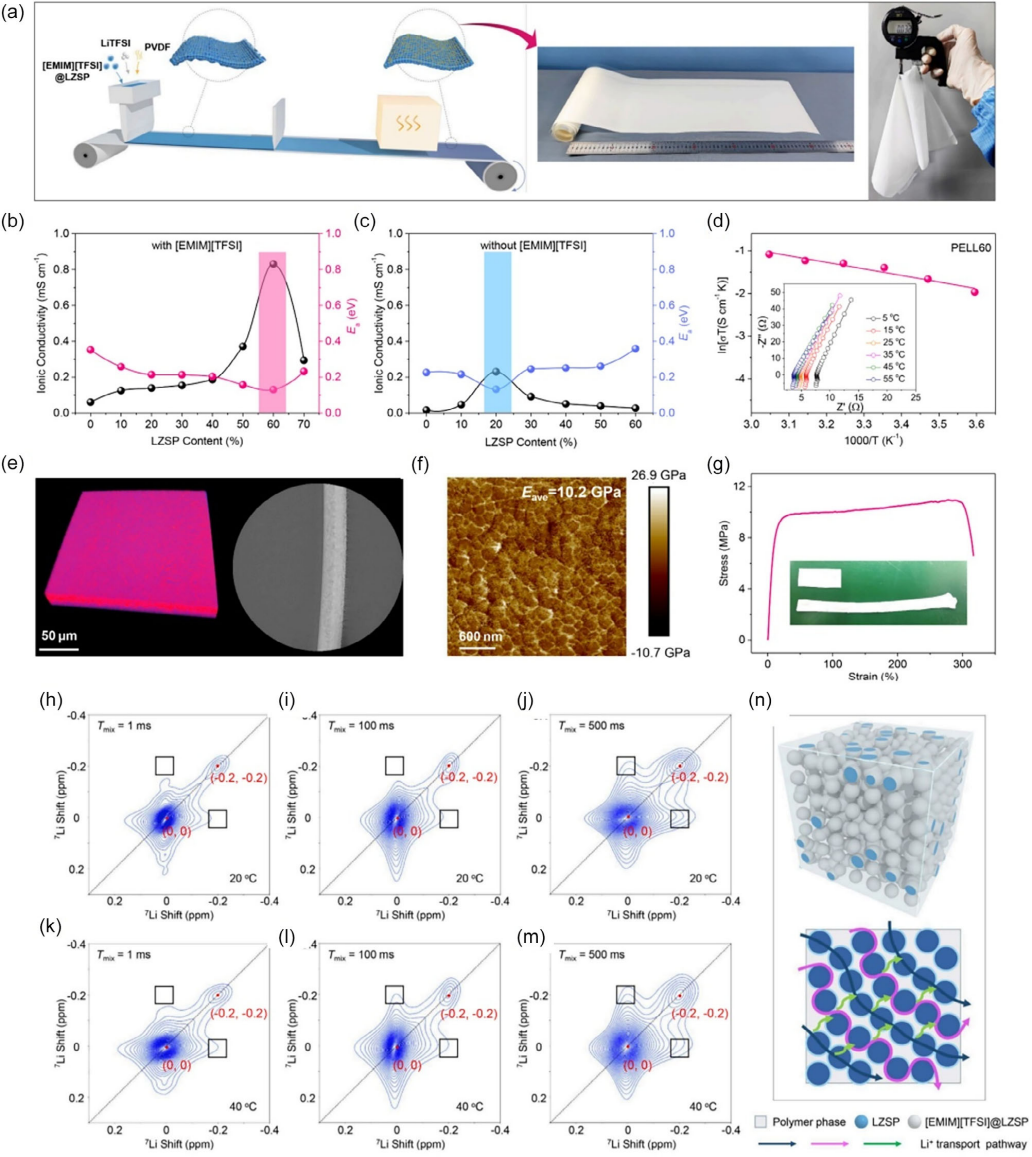
Effect of polymer compatible ionic liquids (PCILs) on Li^+^ conduction mechanism in PVDF/LZSP composite electrolytes. (a) Fabrication of the composite electrolyte, (b) and (c) ionic conductivities and activation energies as a function of the wt% of LZSP at 25 °C with and without PCIL, respectively, (d) EIS profiles at different temperatures and the Arrhenius plot of PELL60, (e) 3D reconstruction collected with XCT, (f) Young's modulus derived from AFM (g) Stress−strain curve, and the digital photo of stretched PELL60. Determination of the Li^+^ ion diffusion pathways across phase boundaries PELL60. (h−m) 2D ^7^Li−^7^Li EXSY spectra of PELL60 at 20 °C (h−j) at 40 °C (k−m), with *T*_mix_ of 1, 100, and 500 ms, respectively, and (n) schematic of the Li^+^ ion diffusion mechanism for PELL60. Reproduced with permission from ref. [221]. Copyright 2024, The American Chemical Society.

In conventional polymer–ceramic composite electrolytes, ceramic fillers often tend to aggregate within the polymer matrix (Figure 12c), which makes it challenging to form continuous and well‐distributed ceramic–polymer interfaces. Also, the intrinsic chemical incompatibility between ceramics and polymers often renders these interfaces inactive for ion transport. To address these issues, a strategy has been proposed in which polymer‐compatible ionic liquids (Figure 12d) (PCILs) are introduced as interfacial mediators between the ceramic fillers and the polymer matrix [221]. In this approach, the polar groups of the PCILs interact with Li^+^ at the ceramic surface while their polymer‐compatible components interact with the surrounding polymer chains. This dual interaction not only improves the dispersion of ceramic particles by mitigating aggregation but also activates the ceramic–polymer interfaces by forming interconnected ion‐transport channels. As a result, Li^+^ can migrate efficiently through the ceramic phase, across the ceramic–polymer interfaces, and through the polymer‐rich regions. The resulting composite electrolyte therefore exhibits a combination of high ionic conductivity, mechanical flexibility, and structural robustness. For example, a composite electrolyte based on PVDF containing 60 wt% PCIL‐coated ((1‐ethyl‐3‐methylimidazolium bis‐(trifluoromethylsulfonyl)imide‐of lithium bis(trifluoromethane‐sulfonyl)‐imide ([EMIM][TFSI])) Li_3_Zr_2_Si_2_PO_12_ (LZSP) (PELL60) fillers demonstrated an ionic conductivity of 0.83 mS cm^−1^, a Li^+^ transference number of 0.81, and an elongation at break of ≈300% at 25 °C (Figure 13) [221]. Notably, this material could be fabricated at the meter scale, and lithium‐metal pouch cells incorporating the electrolyte achieved an energy density of 424.9 Wh kg^−1^ (excluding packaging films) while maintaining puncture resistance.

**FIGURE 14:**
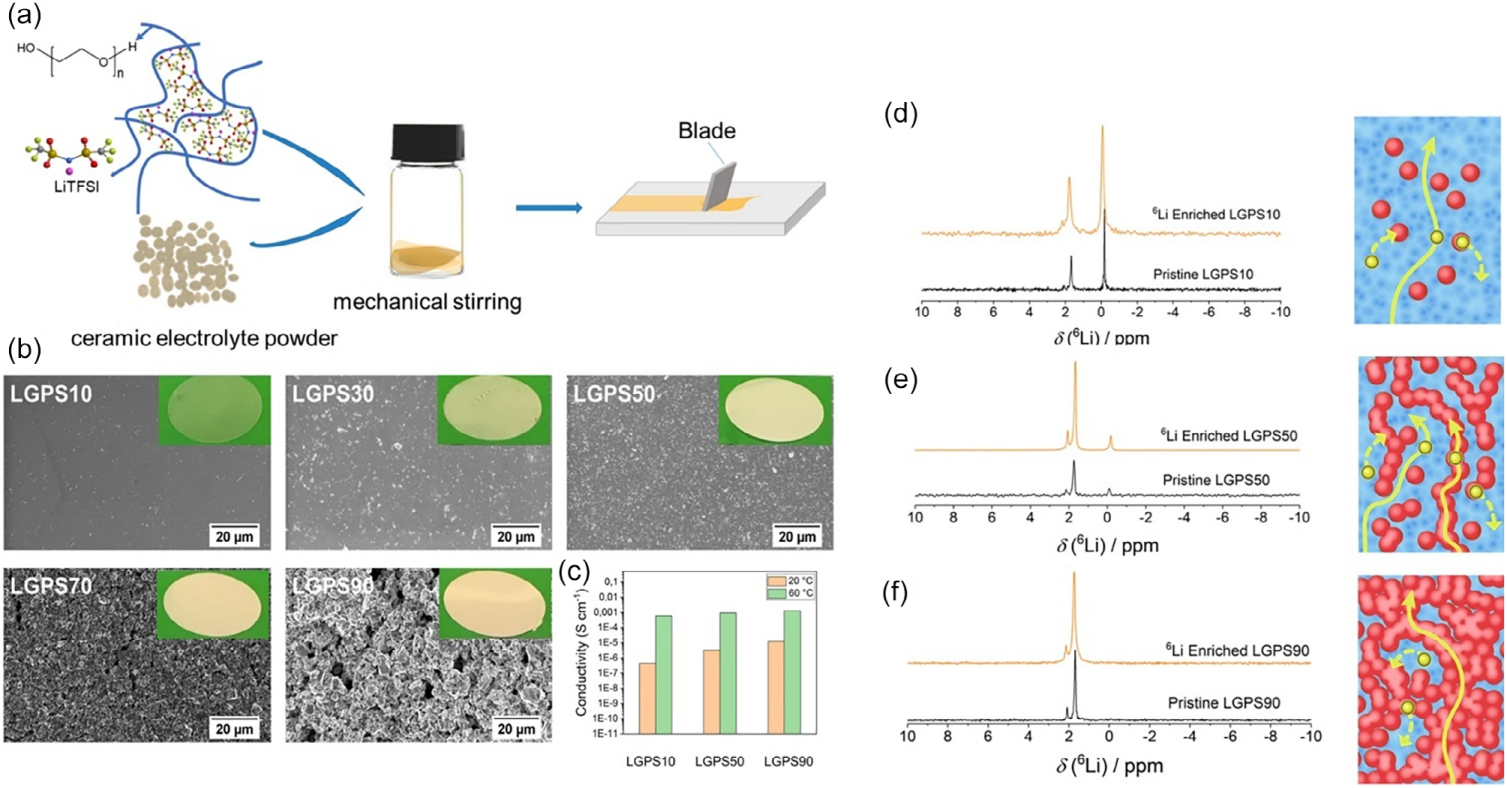
Effect of polymer‐to‐ceramic filler ratio. (a) Preparation of PEO/Li_10_GeP_2_S_12_ (LGPS) composite electrolyte, (b) SEM and the corresponding optical images (insets) of the composite electrolytes with different PEO‐to‐LGPS ratios, (c) ionic conductivities of the composite electrolytes, and ^6^Li‐MAS NMR spectra of (d) LGPS10, (e) LGPS50, and (f) LGPS90, and the respective spectra after cycling against ^6^Li metal electrodes. The illustrative image adjacent to each NMR spectrum depicts a possible Li^+^ transport mechanism in the corresponding composite electrolyte. The LGPS are shown as red spheres, and the blue represents the PEO polymer. Li^+^ ions are represented as yellow spheres. The solid and dotted yellow arrows indicate the movement of Li^+^ within the bulk and at the interface of the SPEs. Reproduced with permission from ref. [222]. Copyright 2021, The American Chemical Society.

In another example, Li et al. reported polymer–sulfide‐modified ceramic composite electrolytes, with particular emphasis on modified ceramic composition‐dependent electrochemical performance and chemical stability [222]. Using polyethylene oxide/Li_10_GeP_2_S_12_ (PEO/LGPS) composites as a model system, the relationship between composition and Li^+^ conduction is systematically investigated (Figure 14). The results reveal distinct changes in transport pathways and electrochemical response as the relative contributions of the polymer and modified ceramic phases are varied, emphasizing the importance of rational composition selection to achieve targeted electrolyte functions. In addition to transport behavior, the chemical stability of the sulfide electrolyte is evaluated under different solvent environments, and potential internal reactions between the polymer and modified ceramic components are considered. These findings highlight the need to consider chemical compatibility during both electrolyte design and processing carefully.

**FIGURE 15:**
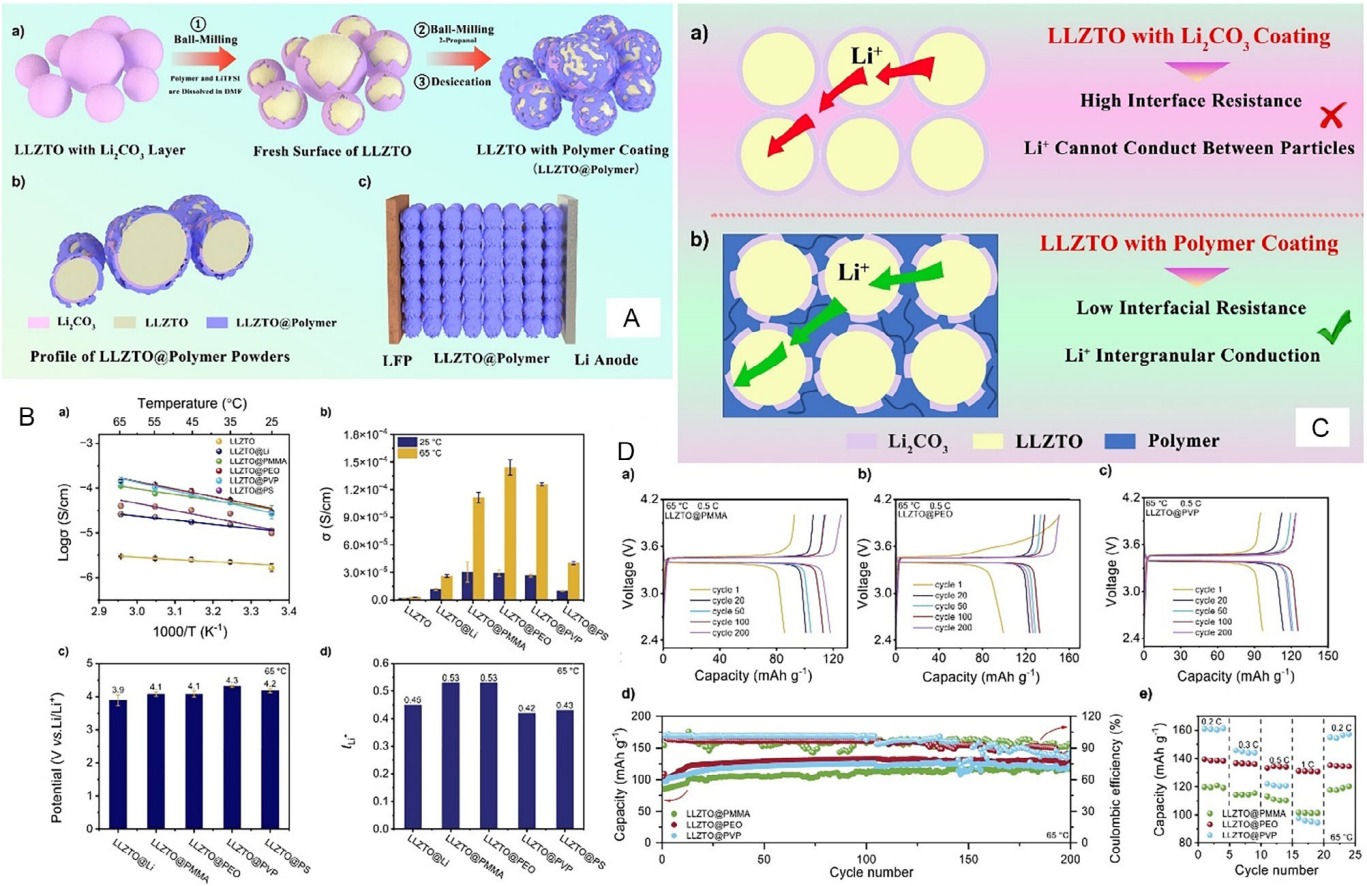
(A) (a) Schematic of the preparation process for LLZTO@polymer composite powders. (b) Cross‐sectional illustration of LLZTO particles coated with polymer, showing partial removal of surface Li_2_CO_3_ layers. (c) Schematic configuration of full solid‐state cells comprising an LFP cathode, Li metal anode, and LLZTO@polymer electrolyte. (B) (a) Arrhenius plots and (b) ionic conductivities at 25 °C and 65 °C for pristine LLZTO, LLZTO@Li, and LLZTO@polymer electrolytes. (c) Electrochemical stability windows and (d) Li^+^ transference numbers (tLi^+^) of LLZTO@Li and LLZTO@polymer electrolytes measured at 65 °C. (C) Schematic illustration of Li^+^ transport mechanisms: (a) pristine LLZTO exhibiting high interfacial resistance due to surface Li_2_CO_3_ layers and interstitial voids, and (b) polymer‐coated LLZTO showing reduced interfacial resistance as a result of Li_2_CO_3_ removal and improved interparticle contact. (D) (a–c) Representative charge–discharge profiles of Li∥LiFePO_4_ full cells employing LLZTO@PMMA, LLZTO@PEO, and LLZTO@PVP electrolytes at 0.5 C. (d) Corresponding discharge capacities and coulombic efficiencies. (e) Rate performance of Li∥LiFePO_4_ full cells with different LLZTO@polymer electrolytes measured at 65 °C. Reproduced with permission from ref. [197]. Copyright 2025, Elsevier.

Recently, Zhang et al. reported a wet‐ball‐milling method to prepare highly conductive garnet‐type Li_6.4_La_3_Zr_1.4_Ta_0.6_O_12_ (LLZTO) solid electrolytes using different polymers [197]. This work employs four representative polymers—PMMA, PEO, PVP, and PS—to produce in situ polymer‐coated LLZTO ceramic particles via a wet ball‐milling process (Figure 15). The resulting polymer coatings enable the construction of continuous lithium‐ion migration pathways between LLZTO particles without high‐temperature sintering, leading to markedly improved electrochemical performance. Lithium symmetric cells exhibit stable cycling for over 2000 h at 65 °C under a current density of 0.1 mA cm^−2^. Furthermore, solid‐state lithium metal batteries assembled with a LiFePO_4_ cathode and the polymer‐coated LLZTO electrolyte deliver a discharge capacity of ≈120 mAh g^−1^, retaining more than 95 % of the initial capacity after 200 charge–discharge cycles at 65 °C and 0.5 C. The enhanced performance is primarily attributed to the in situ formation of polymeric protective layers on freshly exposed LLZTO surfaces during wet ball milling. These coatings suppress surface contamination, improve interparticle contact, and effectively fill voids between adjacent LLZTO particles, thereby facilitating lithium‐ion transport across particle interfaces.

**FIGURE 16:**
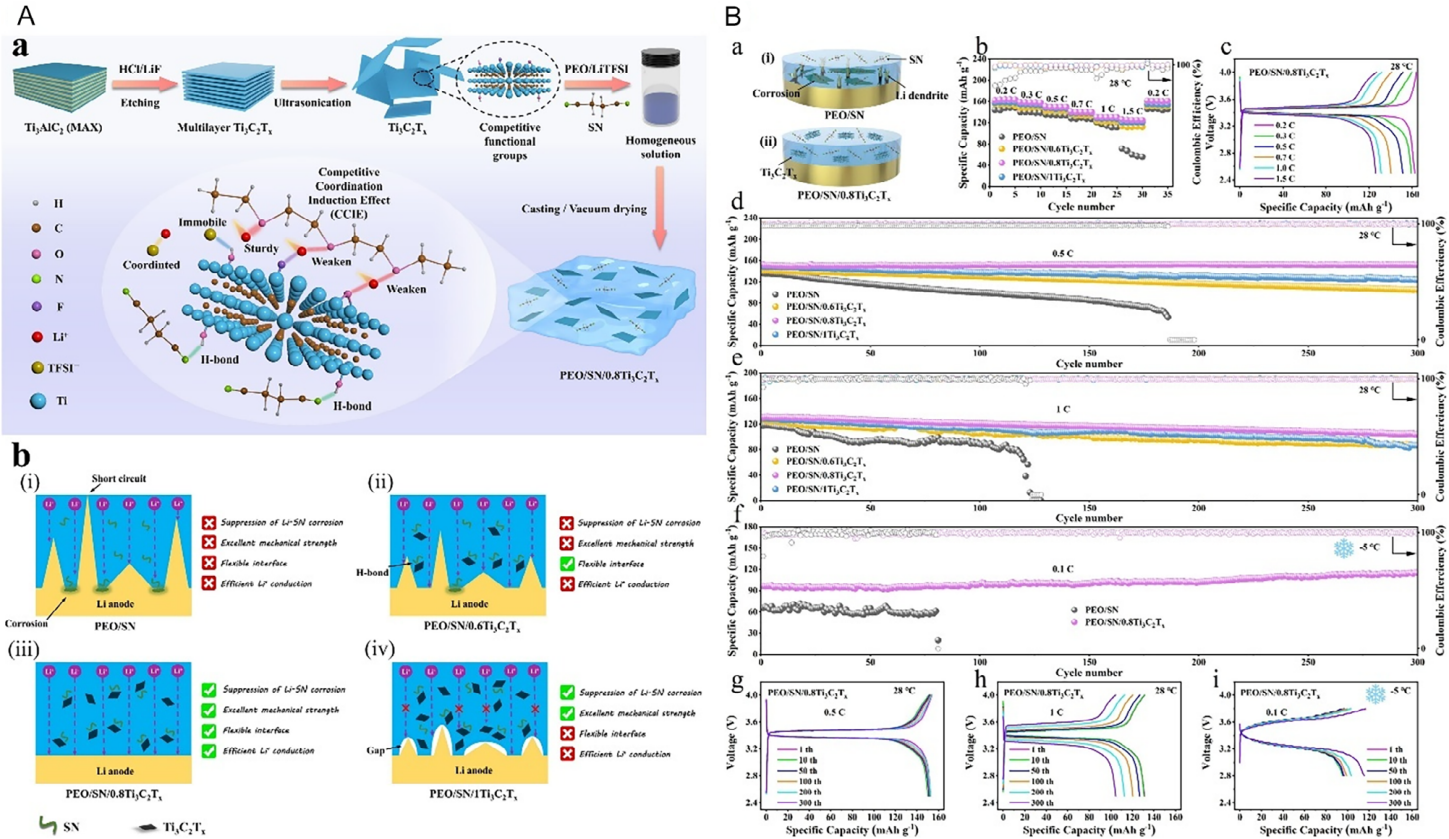
(A) (a) Schematic illustration of the synthesis of solid‐state polymer electrolytes incorporating Ti_3_C_2_T_*x*_ and the corresponding enhancement mechanisms. (b) Schematic comparison of lithium dendrite growth behavior in different solid‐state lithium metal battery configurations. (B) (a) Schematic depiction of Li–electrolyte interphase stability in the absence and presence of Ti_3_C_2_T_*x*_. (b) Rate capabilities of LFP∥PEO/SN∥Li, LFP∥PEO/SN/0.6Ti_3_C_2_T_*x*_∥Li, LFP∥PEO/SN/0.8Ti_3_C_2_T_*x*_∥Li, and LFP∥PEO/SN/1Ti_3_C_2_T_*x*_∥Li full cells measured from 0.2 C to 1.5 C. (c) Representative charge–discharge profiles of the LFP∥PEO/SN/0.8Ti_3_C_2_T_*x*_∥Li full cell at 28 °C. (c) Cycling performance of LFP∥PEO/SN∥Li, LFP∥PEO/SN/0.6Ti_3_C_2_T_*x*_∥Li, LFP∥PEO/SN/0.8Ti_3_C_2_T_*x*_∥Li, and LFP∥PEO/SN/1Ti_3_C_2_T_*x*_∥Li full cells at (d) 0.5 C and (e) 1 C at 28 °C, and (f) 0.1 C at −5 °C (g) 0.5 C and (h) 1 C at 28 °C, and (i) 0.1 C at −5 °C. Reproduced with permission from ref. [203]. Copyright 2025, Elsevier.

On the other hand, SPEs incorporating MXenes are another emerging research theme for the potential realization of next‐generation solid batteries. Several recent studies have shown that the performance of SPEs is enhanced by MXenes by several orders of magnitude. Notably, a SPE incorporating Ti_3_C_2_T_*x*_ is reported by Xu et al., exhibiting a high ionic conductivity of 2.17 × 10^−3^ S cm^−1^ at 35 °C [203]. The presence of Ti_3_C_2_T_*x*_ effectively anchors succinonitrile (SN) molecules in PEO matrix, thereby mitigating corrosion of the lithium metal anode (Figure 16). In parallel, the introduction of competitive coordination interactions weakens the Li^+^ solvation environment within the polymer matrix, facilitating more efficient lithium‐ion transport. By optimizing the Ti_3_C_2_T_*x*_ content, the mechanical properties of the electrolyte are further enhanced, which helps suppress lithium dendrite growth. As a result, Li∥Li symmetric cells demonstrate stable cycling for up to 8000 h at 28 °C. Moreover, LiFePO_4_∥Li full cells deliver a high discharge capacity of 151.7 mAh g^−1^, maintaining 99.3 % capacity retention after 300 cycles. This work not only introduces an effective approach to suppress lithium anode corrosion through succinonitrile stabilization but also provides a simple, scalable, and practical strategy for developing high‐performance SPEs for lithium metal batteries.

Therefore, polymer–filler composite solid electrolytes combine the processability and interfacial compliance of polymers with the mechanical rigidity and, in some cases, fast ion transport characteristics of inorganic or functional fillers. Across a wide range of systems, performance improvements are primarily governed not by filler content alone but by polymer–filler interfacial chemistry, dispersion quality, and connectivity of ion‐transport pathways. Properly engineered fillers can suppress polymer crystallinity, enhance mechanical stability, and reduce interfacial resistance, while poorly integrated fillers often introduce transport bottlenecks. Recent studies demonstrate that surface‐modified ceramics, 2D fillers, and in situ‐formed polymer coatings are particularly effective in improving both ionic conductivity and cycling stability. Nevertheless, challenges remain in achieving reproducible filler dispersion, maintaining room‐temperature conductivity, and ensuring long‐term interfacial stability with lithium metal and high‐voltage cathodes. Continued progress will depend on interface‐focused design strategies and realistic electrochemical benchmarking rather than increasing composite complexity.

Although several mechanisms have been proposed to explain the conductivity enhancement observed in polymer–ceramic composite electrolytes, the level of experimental evidence supporting each mechanism varies considerably. The suppression of polymer crystallinity and the promotion of lithium‐salt dissociation through Lewis acid–base interactions are well supported by spectroscopic and electrochemical studies. In contrast, mechanisms involving space‐charge conduction, continuous ceramic‐ion percolation, and preferential interfacial Li^+^ transport remain more system‐dependent and have not been fully demonstrated. Their relative contributions depend strongly on filler composition, particle size, surface chemistry, dispersion, and polymer–ceramic interfacial compatibility. Recent work by Wang et al. [197] demonstrated that MOF fillers containing abundant Lewis acid sites establish continuous MOF/polymer interfaces that enhance LiTFSI dissociation and create interconnected Li^+^ transport pathways, providing direct experimental evidence that interfacial chemistry, rather than filler incorporation alone, governs conductivity enhancement. These findings suggest that future composite electrolyte design should prioritize interface engineering and quantitative mechanistic validation rather than relying solely on increased ceramic loading.

## 4 SPEs Beyond LIBs

### 4.1 Lithium–Sulfur (Li–S) and Lithium‐Oxygen (Li–O_2_) Batteries

Beyond conventional LIBs, SPEs have also emerged as promising candidates for high‐energy‐density battery systems, particularly lithium–sulfur (Li–S) [223, 224, 225] and lithium–oxygen (Li–O_2_) [226, 227] batteries. These specialized chemistries offer theoretical energy densities that substantially exceed those of conventional LIBs but are hindered by severe interfacial instability, electrolyte degradation, and poor cycling reversibility [228, 229]. The inherent safety, mechanical flexibility, and interfacial conformity of SPEs provide opportunities to mitigate several of these limitations while enabling the use of lithium metal anodes [230]. In Li–S batteries, the dissolution and migration of lithium polysulfides between the sulfur cathode and lithium anode, commonly known as the polysulfide shuttle effect, lead to rapid capacity fading, low Coulombic efficiency, and accelerated degradation of the lithium metal surface. SPEs provide an effective physical barrier that suppresses polysulfide diffusion while maintaining lithium‐ion transport between the electrodes [231, 232]. In addition, polymer electrolytes improve interfacial contact with lithium metal, reducing dendrite growth and enhancing cycling stability. Despite these advances, practical Li–S batteries continue to face challenges associated with limited room‐temperature ionic conductivity, interfacial resistance, and maintaining stable cycling under lean‐electrolyte and high‐sulfur‐loading conditions [233, 234, 235].

In conventional Li–O_2_ cells, liquid electrolytes undergo continuous decomposition due to reactions with superoxide intermediates, resulting in poor cycle life and electrolyte instability. Polymer electrolytes provide improved chemical stability and suppress electrolyte evaporation while maintaining intimate contact with the lithium metal anode [226, 236, 237]. Nevertheless, Li–O_2_ batteries present additional challenges that differ fundamentally from those of LIBs. Oxygen transport through the electrolyte, formation and decomposition of lithium peroxide (Li_2_O_2_), electrolyte oxidation at high charging potentials, and accumulation of insulating discharge products all limit practical performance. Consequently, recent research has focused on polymer electrolytes possessing high oxidative stability, controlled oxygen permeability, and stable electrode–electrolyte interfaces [238, 239, 240]. Composite polymer electrolytes containing ceramic fillers and catalytic nanoparticles have shown promising improvements in ionic conductivity and interfacial stability while facilitating more reversible oxygen electrochemistry [241]. Although Li–S and Li–O_2_ batteries require electrolyte designs tailored to their distinct electrochemical reactions, many of the fundamental design principles developed for lithium‐ion SPEs—including interface engineering, polymer architecture optimization, composite electrolyte design, and dendrite suppression—remain directly applicable. Continued integration of high‐conductivity polymer matrices with multifunctional fillers and stable interfacial chemistries is expected to accelerate the practical development of these next‐generation high‐energy battery systems.

### 4.2 Sodium‐Ion, Potassium‐Ion, and Zinc‐Ion Batteries

Recently, various material design strategies have been reported for the fabrication of SPEs intended for use in postlithium battery technologies [242, 243, 244, 245]. These systems are being actively investigated to address concerns regarding lithium resource availability, cost, and sustainability while enabling application‐specific energy‐storage solutions. However, the different ionic radii, charge densities, electrochemical potentials, and interfacial chemistries of Na^+^ [246, 247, 248], K^+^ [249, 250], Zn^2+^ [251, 252], and multivalent ions [253] impose distinct electrolyte requirements compared with lithium‐based systems. Among these technologies, solid‐state sodium‐ion batteries (SSSBs) have attracted considerable attention because of the natural abundance and low cost of sodium resources [254]. Polymer electrolytes based on PEO, polycarbonates, and gel–polymer systems have demonstrated encouraging sodium‐ion transport; however, the larger ionic radius and stronger ion–polymer interactions of Na^+^ generally result in lower ionic conductivity and slower diffusion kinetics than lithium systems [255, 256]. Consequently, polymer architectures with higher segmental mobility, lower glass‐transition temperatures, and optimized sodium‐salt dissociation are being actively explored. PIBs offer another attractive alternative because potassium exhibits a relatively low redox potential and fast ion transport in suitable electrolytes [257, 258]. Nevertheless, the larger K^+^ ion presents additional challenges for polymer electrolytes, including reduced salt dissociation efficiency, increased polymer coordination strength, and lower mechanical stability during repeated cycling. Recent efforts have therefore focused on soft polymer matrices, highly dissociative potassium salts, and composite polymer electrolytes to facilitate potassium‐ion transport [257, 259]. For aqueous and quasisolid‐state zinc‐ion batteries (ZIBs), polymer electrolytes primarily function as ion‐conducting membranes while suppressing water evaporation, zinc dendrite formation, and parasitic side reactions [260, 261]. Hydrophilic polymers such as PVA, PAM, PEG, and cellulose derivatives have shown considerable promise because they regulate Zn^2+^ solvation structures while maintaining high ionic conductivity and mechanical flexibility. Unlike lithium systems, electrolyte design for zinc batteries must also balance ionic transport with water management and electrochemical stability.

## 5 Manufacturability and Manufacturing Metrics for Industrial‐Scale SPEs

Although laboratory‐scale fabrication methods such as solution casting, hot pressing, electrospinning, and in situ polymerization have demonstrated considerable success, the commercial implementation of SPEs ultimately depends on their compatibility with continuous manufacturing processes. Roll‐to‐roll (R2R) coating has emerged as one of the most promising production technologies because it enables high‐throughput fabrication of thin electrolyte films [262] with controlled thickness, reduced manufacturing cost, and compatibility with existing LIB production lines [263, 264, 265]. Among the critical manufacturing parameters, coating speed directly determines production throughput and manufacturing cost. However, increasing coating speed often alters slurry flow behavior, drying kinetics, and film formation, which can lead to coating defects and thickness nonuniformity [266]. Consequently, optimizing the relationship between coating speed, drying conditions, and electrolyte quality remains a major engineering challenge for large‐scale manufacturing. Another important manufacturing metric is electrolyte thickness uniformity, which is commonly quantified by the coefficient of variation across the coated web [267]. Thickness variations result in localized current‐density fluctuations, nonuniform lithium deposition, and increased interfacial resistance, thereby reducing cell reliability [268]. Maintaining highly uniform coatings over meter‐scale substrates is therefore essential for commercial SSB production. Likewise, defect density, including pinholes, cracks, particle agglomeration, coating discontinuities, and voids, significantly influences battery performance and safety. Even isolated defects may generate local current hotspots that accelerate lithium dendrite penetration or mechanical failure during repeated cycling. Consequently, defect inspection and process control have become indispensable components of industrial battery manufacturing. For solution‐based processing [269], solvent residue is another critical parameter because incomplete solvent removal may plasticize the polymer matrix, modify ionic conductivity, reduce electrochemical stability, and compromise adhesion at electrode–electrolyte interfaces. Careful optimization of solvent evaporation, drying temperature, residence time, and web speed is therefore required to achieve reproducible electrolyte performance. Therefore, the future development of SPEs should optimize manufacturing robustness alongside electrochemical performance. In addition to ionic conductivity and electrochemical stability, practical SPEs should be evaluated using manufacturing metrics such as coating throughput, thickness uniformity, defect density, residual solvent content, and process reproducibility, as these factors ultimately determine industrial scalability and commercialization.

## 6 Safety Aspects of SPEs

Safety considerations are a major driver behind the development of SSB systems, and SPEs are often viewed as a practical alternative to conventional liquid electrolytes in this context [270]. The absence of free liquid components significantly reduces risks associated with leakage and flammability, which are well‐known issues in current LIBs [52, 271]. In addition, the solid yet compliant nature of polymer electrolytes allows for improved contact with electrodes, which can contribute to more stable interfacial behavior during cycling. The following sections discuss the thermal, electrochemical, and mechanical aspects of safety in SPE‐based systems, with a focus on dendrite suppression and tolerance to abuse conditions.

### 6.1 Thermal and Electrochemical Safety

A key safety‐related advantage of SPEs is their limited flammability compared with organic liquid electrolytes. Because SPEs do not rely on low‐boiling organic solvents, the likelihood of ignition or rapid combustion at elevated temperatures is reduced. Thermal analysis studies generally show that polymer electrolytes decompose at higher temperatures than liquid electrolyte systems, although the exact behavior depends on the polymer backbone, lithium salt, and the presence of plasticizers or additives. From a practical standpoint, this improved thermal stability can delay the onset of thermal runaway and reduce the severity of failure events [272]. Thermal shrinkage is another important factor influencing cell safety [273]. Conventional polyolefin separators used in liquid‐electrolyte cells can shrink significantly when exposed to high temperatures, which may lead to internal short circuits. In contrast, SPEs typically exhibit better dimensional stability, particularly in cross‐linked or composite formulations [274, 275]. The resistance to shrinkage helps maintain physical separation between electrodes during thermal excursions and contributes to more predictable cell behavior under stress [276]. Beyond replacing flammable liquid electrolytes, recent research has focused on developing intrinsically nonflammable polymer electrolyte systems through rational molecular and structural design. Rather than relying solely on flame‐retardant additives, these strategies integrate fire‐resistant components directly into the electrolyte architecture while preserving ionic conductivity and interfacial stability. For example, Tan et al. [277] developed an asymmetric fire‐retardant quasi‐solid electrolyte that combines a flame‐retardant polymer framework with asymmetric electrolyte engineering to suppress thermal runaway while maintaining stable lithium‐ion transport and electrode compatibility. This work demonstrates that electrolyte safety can be achieved through synergistic optimization of polymer chemistry, interfacial stability, and electrolyte architecture rather than through conductivity enhancement alone. Such design strategies provide an important direction for the development of practical solid‐state batteries with improved safety and manufacturability.

Electrochemical stability is closely linked to safety, especially when SPEs are paired with high‐voltage cathode materials. Many polymer electrolytes show reasonably wide electrochemical stability windows, limiting oxidative decomposition and gas formation during operation [278, 279]. However, interfacial reactions can still occur at elevated potentials, and long‐term stability remains dependent on electrolyte composition and interface chemistry. As a result, electrochemical safety cannot be considered independently of interfacial design.

### 6.2 Dendrite Suppression

Lithium dendrite growth is a critical safety concern for batteries employing lithium‐metal anodes, and the mechanical properties of SPEs strongly influence this process. Compared with liquid electrolytes, SPEs provide a continuous solid medium that can mechanically resist dendrite penetration [110]. Several studies have suggested that electrolytes with higher elastic modulus are more effective at limiting dendrite propagation, particularly under moderate current densities [280, 281, 282]. At the same time, polymer elasticity plays an important role in maintaining stable interfaces during cycling. Polymer electrolytes that are too rigid may suffer from poor interfacial contact as lithium undergoes repeated plating and stripping, leading to increased interfacial resistance and nonuniform current distribution. Softer or viscoelastic polymers can accommodate volume changes more effectively, helping to preserve contact at the lithium–electrolyte interface. Achieving an appropriate balance between mechanical stiffness and elasticity is therefore essential for both dendrite suppression and long‐term cycling stability. The use of composite polymer electrolytes has been widely explored as a means of improving dendrite resistance [283, 284]. Incorporating inorganic fillers can increase the local mechanical strength of the electrolyte while retaining overall flexibility [285, 286]. Although such approaches have shown promise, their effectiveness often depends on filler dispersion, interfacial compatibility, and operating conditions, and dendrite suppression under high current densities remains challenging.

### 6.3 Abuse Tolerance

Under overcharging conditions, SPE‐based cells generally exhibit reduced gas evolution and slower heat generation compared with liquid‐electrolyte systems [287]. The lack of volatile components limits pressure buildup, although electrolyte decomposition and interfacial degradation can still occur at high potentials [288]. Mechanical abuse, such as bending, compression, or vibration, is another area where polymer electrolytes offer advantages [289, 290]. Unlike brittle ceramic electrolytes, SPEs can tolerate a certain degree of deformation without cracking, which helps prevent sudden internal short circuits. This mechanical compliance is particularly relevant for applications involving flexible cells or batteries exposed to mechanical stress during operation [291]. Overall, SPEs provide clear safety benefits compared with conventional liquid electrolytes, but their performance under extreme thermal, electrical, and mechanical conditions remains closely tied to material composition and interface design. Continued efforts to improve mechanical robustness, interfacial stability, and electrochemical durability are required to fully realize the safety potential of SPE‐based solid‐state batteries.

## 7 Sustainability and Environmental Considerations for SPEs

Sustainability has become an increasingly important criterion in the development of next‐generation battery technologies, particularly as large‐scale deployment in EVs and stationary energy storage accelerates. While SPEs are often discussed primarily in the context of safety and electrochemical performance, their environmental impact across the full life cycle is also an important factor [99, 292]. Compared with conventional liquid electrolytes, SPEs offer certain sustainability‐related advantages, including the potential for solvent‐free processing and reduced reliance on volatile organic compounds [293]. However, these benefits are not universal and depend strongly on material selection, synthesis routes, and cell design.

### 7.1 Material Selection and Resource Considerations

The sustainability of SPEs is closely linked to the choice of polymer host and lithium salt. Many widely studied SPEs are based on synthetic polymers derived from petrochemical feedstocks, which raise concerns regarding resource availability and environmental footprint. In addition, commonly used lithium salts often contain fluorinated anions, which can present challenges related to environmental persistence and end‐of‐life handling. Recent research has explored alternative polymer matrices derived from renewable or bio‐based sources, such as cellulose derivatives, chitosan, and other polysaccharides [294, 295, 296]. These materials offer the potential to reduce dependence on fossil‐based resources and may improve biodegradability under appropriate conditions [242]. Nevertheless, their electrochemical performance and long‐term stability generally lag behind those of conventional synthetic polymers, and further optimization is required before such materials can be considered viable for practical battery applications. Salt chemistry also plays a critical role in sustainability. The development of fluorine‐free lithium salts has gained attention as a strategy to reduce environmental impact and simplify recycling [297]. While some of these salts show promising electrochemical properties, their compatibility with polymer hosts and their stability under high‐voltage operation remain active areas of investigation.

### 7.2 Manufacturing and Processing Considerations

Manufacturing processes have a substantial influence on the overall environmental footprint of polymer electrolyte‐based batteries. One potential advantage of SPEs is their compatibility with solvent‐free or low‐solvent processing techniques, such as melt processing or in situ polymerization [293]. These approaches can reduce solvent use, lower energy consumption, and simplify electrolyte fabrication compared with liquid electrolyte systems. However, many high‐performance SPE formulations still rely on organic solvents during synthesis or membrane casting, which can offset some of the anticipated environmental benefits. The use of cross‐linking agents, plasticizers, and inorganic fillers further complicates processing and may introduce additional environmental and safety considerations. From a scalability perspective, developing SPE systems that combine acceptable electrochemical performance with simple, low‐energy manufacturing routes remains a key challenge. In addition, the integration of SPEs into full cells must be considered. Processing conditions compatible with polymer electrolytes, such as moderate temperatures and pressures, can be advantageous for large‐scale production. At the same time, achieving uniform electrolyte layers and stable interfaces in practical cell configurations requires careful control of fabrication parameters.

### 7.3 Recyclability and End‐of‐Life Considerations

End‐of‐life management is an important but often overlooked aspect of sustainability in SSB systems [298]. The solid nature of polymer electrolytes offers potential advantages for recycling, as it eliminates liquid electrolyte leakage and may simplify cell disassembly [299]. In principle, polymer electrolytes could be separated mechanically from electrodes, reducing contamination during recycling processes. Despite this potential, the recyclability of SPE‐based batteries is not yet well established. Cross‐linked polymers and composite electrolytes containing inorganic fillers can be difficult to separate and process at the end of life. In addition, the chemical stability that is desirable during battery operation may hinder degradation or recycling under standard conditions [300]. Designing polymer electrolytes that balance durability with recyclability remains an unresolved issue. Emerging concepts, such as recyclable polymer networks or electrolytes designed for controlled depolymerization, offer possible pathways toward more sustainable SSB architectures [301, 302]. However, these approaches are still at an early stage and require further validation in realistic battery systems.

Overall, SPEs present both opportunities and challenges from a sustainability point of view. While they offer clear advantages in terms of safety and the potential for solvent‐free processing, their environmental impact depends strongly on material chemistry and manufacturing choices. Progress toward truly sustainable SPE‐based batteries will require coordinated advances in polymer design, salt chemistry, processing methods, and recycling strategies. Continued efforts to evaluate sustainability alongside electrochemical performance are essential to ensure that SPEs contribute meaningfully to the development of environmentally responsible energy storage technologies.

## 8 Practical Challenges in SPEs

This section outlines the key practical challenges that currently hinder the widespread adoption of SPEs in commercial battery systems. Despite continued research and substantial progress at the laboratory scale, the commercialization of polymer‐based solid‐state batteries remains limited [303]. Bridging the gap between promising experimental results and practical deployment requires addressing a combination of material limitations, interfacial challenges, and manufacturing constraints. In contrast to conventional liquid electrolytes, SPEs must simultaneously provide high ionic conductivity, mechanical robustness, interfacial stability, and scalability.

### 8.1 Limited Ionic Conductivity at Ambient Conditions

Low ionic conductivity at room temperature remains one of the most significant obstacles to the practical application of SPEs. While liquid electrolytes typically exhibit ionic conductivities on the order of 10^−2^ S cm^−1^, conventional polymer electrolytes—particularly those based on PEO—generally achieve conductivities in the range of 10^−5^ to 10^−7^ S cm^−1^ under ambient conditions [304]. This difference arises from the fundamental ion‐transport mechanism in polymers, which is strongly linked to the segmental motion of polymer chains and therefore highly temperature‐dependent. Below the glass transition temperature of the polymer, segmental mobility is restricted, leading to slow ion transport. As a result, acceptable ionic conductivity is often achieved only at elevated temperatures, typically above 60 °C, which limits the applicability of SPEs in many real‐world scenarios. Various strategies, including polymer blending, plasticization, copolymerization, and the incorporation of inorganic fillers, have been explored to enhance room‐temperature conductivity [305, 306]. However, these approaches frequently involve trade‐offs, such as reduced mechanical strength, narrower electrochemical stability windows, or increased complexity in materials processing. Achieving high ionic conductivity without compromising other essential properties remains an unresolved challenge.

### 8.2 Electrode–Electrolyte Interfacial Instability

Interfacial stability between SPEs and electrodes is another critical limitation in SSB systems [307, 308]. Unlike liquid electrolytes, which can readily wet porous electrodes, SPEs often suffer from imperfect physical contact at the electrode–electrolyte interface [309]. This can lead to void formation, increased interfacial resistance, and nonuniform lithium‐ion flux during cycling [310]. In lithium‐metal batteries, chemical interactions between the polymer electrolyte and the highly reactive lithium surface can further worsen interfacial degradation [311]. Side reactions at the interface may result in the formation of resistive interphases, increasing overpotential, and accelerating capacity fade. Mechanical stresses generated during repeated lithium plating and stripping can also disrupt interfacial contact, leading to delamination or localized current hotspots [281]. Although interface‐modified electrolytes and artificial interlayers have shown promise in mitigating some of these effects, achieving a chemically and mechanically stable interface [312] over long cycle life remains a significant challenge.

### 8.3 System Integration and Real‐World Performance

Beyond material‐level challenges, system‐level integration represents a final barrier to the deployment of SPEs in commercial batteries. In practical applications, such as EVs and grid‐scale energy storage, batteries must operate reliably over thousands of cycles while experiencing variations in temperature, pressure, and current density. SPEs must therefore maintain stable ionic conductivity, mechanical properties, and interfacial integrity throughout their extended operation. Aging phenomena, including polymer degradation, interfacial resistance growth, and loss of electrode contact, can lead to irreversible performance decay over time. Importantly, many reported studies rely on idealized laboratory testing conditions that do not fully reflect the complexities of real‐world operation. More extensive testing under realistic conditions is required to accurately assess the durability, safety, and failure modes of SPE‐based solid‐state batteries. In addition, the lack of standardized testing protocols and cell configurations further complicates direct comparison between studies and slows technological maturation.

## 9 Socioeconomic Impact of SPEs Integrated Batteries

The development of SPEs has implications that extend beyond materials science and electrochemical performance, influencing broader economic, industrial, and societal dimensions of energy storage technologies [313]. As demand for safer and more sustainable batteries increases, particularly in transportation and stationary energy storage, the potential socioeconomic impact of polymer‐based solid‐state batteries warrants careful consideration. While SPEs offer promising technical advantages, their large‐scale adoption will depend not only on performance improvements but also on cost, manufacturability, and compatibility with existing industrial infrastructures.

### 9.1 Cost Considerations and Economic Viability

Cost remains a decisive factor in determining the commercial feasibility of any battery technology. Although SPEs can reduce certain safety‐related costs by eliminating flammable liquid components and complex containment systems, their current material and processing costs are often higher than those of conventional liquid electrolytes. High‐purity polymers, specialty lithium salts, and controlled manufacturing environments contribute significantly to overall system cost, particularly at early stages of development [314]. In the long term, SPEs may offer economic benefits through simplified cell architecture and reduced requirements for safety management and thermal control [315]. Polymer electrolytes also have the potential to enable thinner separators and more compact cell designs, which could improve energy density at the pack level. However, these advantages are highly dependent on achieving scalable production routes and consistent performance, and their economic impact remains uncertain until large‐scale manufacturing is demonstrated [316].

### 9.2 Industrial Transition and Manufacturing Infrastructure

From an industrial perspective, the transition from conventional battery manufacturing to a process that considers incorporating SPEs in place of liquid electrolytes presents both challenges and opportunities [317]. On the one hand, capital investment and process re‐engineering could slow adoption, particularly in cost‐sensitive markets. On the other hand, the emergence of SSB technologies could stimulate innovation across the supply chain, creating opportunities for new manufacturers, material suppliers, and processing technologies [318]. The extent to which SPEs can be integrated into existing manufacturing ecosystems will strongly influence their socioeconomic impact.

### 9.3 Supply Chain and Resource Implications

SPEs may also affect the battery supply chain by altering material demand and sourcing strategies [318, 319]. Compared with liquid electrolytes, SPEs can reduce reliance on volatile organic solvents and, in some cases, simplify electrolyte handling and transport. The use of alternative polymer hosts or fluorine‐free lithium salts could further reduce dependence on critical or environmentally problematic materials. At the same time, many polymer electrolytes still rely on petrochemical feedstocks or specialized additives, which introduces its own resource and sustainability considerations. The development of bio‐based polymers and more environmentally benign salts has the potential to diversify supply chains and reduce geopolitical risks associated with critical materials. However, these approaches must be evaluated in terms of scalability, performance, and cost to assess their real socioeconomic impact.

### 9.4 Safety, Regulation, and Public Acceptance

Safety improvements associated with SPE‐based batteries could have important socioeconomic implications, particularly in applications where battery failure carries high risk. Enhanced safety may reduce regulatory barriers, lower insurance and compliance costs, and improve public acceptance of battery technologies in EVs, residential energy storage, and densely populated environments [313]. In this context, SPEs could contribute to broader adoption of electrified systems by addressing safety concerns that limit deployment [320]. However, regulatory frameworks for solid‐state batteries are still evolving, and standardized testing and certification protocols remain limited. The lack of clear regulatory pathways may delay market entry and increase development costs. Establishing consistent safety standards and performance benchmarks will be essential to realizing the potential socioeconomic benefits of SPE‐based systems.

### 9.5 Workforce and Technological Development

Polymer electrolyte technologies draw on expertise from polymer chemistry, materials science, electrochemistry, and manufacturing engineering, potentially encouraging interdisciplinary collaboration and workforce diversification. As the technology matures, demand for specialized skills in polymer processing and solid‐state device fabrication may increase. At the same time, the pace of technological change could create challenges for workforce retraining and industrial adaptation. Ensuring that knowledge transfer and workforce development keep pace with technological advances will be important for maximizing the positive socioeconomic impact of SPE‐based battery technologies.

On the whole, SPEs have the potential to influence the socioeconomic landscape of energy storage by improving safety, enabling new battery architectures, and reshaping manufacturing and supply chains. However, these impacts will depend on overcoming existing technical and economic barriers and on the successful integration of SPEs into large‐scale production systems [321]. A balanced assessment of performance, cost, safety, and manufacturability will be necessary to determine the extent to which SPEs can contribute to economically viable and socially beneficial energy storage solutions [322].

## 10 Summary and Outlook

SPEs have attracted significant attention as alternatives to conventional liquid electrolytes for advanced energy storage systems owing to their enhanced safety, mechanical flexibility, and adaptability in cell design [323, 324]. By eliminating volatile and flammable liquid components, SPEs address several other intrinsic limitations of liquid electrolytes, including leakage, thermal instability, and safety concerns under abusive conditions [50]. As a result, polymer‐based electrolytes are increasingly explored for use in lithium‐metal and emerging postlithium battery systems, where safety and long‐term stability are critical requirements [325, 326]. At a fundamental level, ion transport in polymer electrolytes is closely linked to the dynamics of the polymer host. Lithium‐ion mobility depends on segmental motion of the polymer chains and on coordination–decoordination processes between the ions and polar functional groups along the backbone. This intrinsic coupling between mechanical and transport properties has highly limited the achievable ionic conductivity, particularly at room temperature. To overcome this limitation, a wide range of material design strategies has been developed, including polymer–ceramic composite electrolytes, block copolymer architectures, single‐ion conducting polymers, and dynamically cross‐linked or self‐healing systems. Among these approaches, polymer–ceramic composite electrolytes have shown particularly promising performance. By incorporating ceramic fillers such as Li_7_La_3_Zr_2_O_12_, Li_1.__3_Al_0.__3_Ti_1.__7_(PO_4_)_3_, or Li_1.__4_Al_0.__4_Ge_1.__6_(PO_4_)_3_ into polymer matrices, it is possible to enhance ionic conductivity while simultaneously improving mechanical strength and thermal stability. High conductivities have been reported in optimized systems, attributed to improved salt dissociation, interfacial ion transport, and the formation of percolated conduction pathways. In addition to transport benefits, ceramic fillers can reinforce the polymer matrix and contribute to resistance against lithium dendrite penetration, which is essential for the safe operation of lithium‐metal batteries. Block copolymer electrolytes provide an alternative route to balancing mechanical and electrochemical requirements. In these systems, chemically distinct blocks form ion‐conducting and mechanically rigid domains through microphase separation. The resulting nanostructures can create continuous pathways for ion transport while maintaining sufficient mechanical integrity. This decoupling of ionic mobility from mechanical stiffness has long been a goal in polymer electrolyte design and continues to motivate research into new block chemistries and morphologies.

SIPEs represent another critical development. By immobilizing the anionic species on the polymer backbone, these materials restrict anion motion and increase the lithium‐ion transference number. This approach reduces concentration polarization during cycling and can improve rate capability and cycling stability, particularly under high current densities. However, achieving high conductivity in single‐ion systems remains challenging, and continued optimization of polymer architecture and ion‐binding motifs is required. More recently, self‐healing polymer electrolytes have been explored as a means of improving their long‐term durability. These materials incorporate reversible interactions, such as dynamic covalent bonds or hydrogen‐bonding networks, which allow the polymer matrix to recover from mechanical damage induced during cycling. While still at an early stage, self‐healing electrolytes offer a potential strategy for mitigating mechanical degradation and extending cell lifetime without sacrificing flexibility or ionic transport.

Despite these advances, the electrode–electrolyte interface remains a critical bottleneck. Poor interfacial contact and chemical incompatibility can lead to high resistance and accelerated degradation. To address this issue, in situ polymerization techniques have been developed, allowing the electrolyte to form directly within porous electrode structures and thereby improving interfacial conformity. The incorporation of ionic liquids into polymer electrolytes has also been investigated as a way to enhance interfacial wettability and suppress unwanted side reactions at lithium surfaces. However, trade‐offs with mechanical strength and stability must be carefully managed. Computational methods are playing an increasingly important role in guiding electrolyte development. MD and DFT simulations provide detailed insight into ion coordination environments, diffusion mechanisms, and interfacial behavior at the atomic scale [95, 327, 328, 329]. More recently, machine learning (ML) approaches have been applied to predict polymer properties and screen large compositional spaces [330, 331]. When combined with experimental validation, these data‐driven methods can significantly accelerate material discovery and optimization. The integration of computational design with automated synthesis and testing is expected to further reduce development time for new polymer electrolyte systems [332].

Although laboratory‐scale progress has been substantial, significant challenges remain in translating these materials into commercial batteries [333]. Manufacturing uniform, defect‐free polymer electrolyte films over large areas, ensuring compatibility with existing battery production processes, and maintaining stable performance over extended cycling are ongoing concerns. Industrial efforts by companies such as Toyota, Solid Power, and QuantumScape have demonstrated prototype solid‐state cells that incorporate polymer‐based or hybrid electrolytes, highlighting both the potential and the remaining limitations of current technologies. From a longer‐term perspective, increasing attention is being directed toward cost, recyclability, and environmental impact. The development of polymer electrolytes based on bio‐derived or more sustainable polymers, including cellulose, chitosan, and certain polycarbonates, may help reduce dependence on petrochemical feedstocks and improve the overall sustainability of solid‐state batteries. However, these materials must still meet stringent performance and durability requirements to be viable in practical applications. Overall, continued progress in SPEs will likely depend on the development of multifunctional hybrid systems that integrate high ionic conductivity, mechanical robustness, and stable interfaces. Advances in polymer chemistry, composite design, and computational tools are expected to play complementary roles in this process. As these efforts converge, SPEs are poised to become a key component of future solid‐state batteries, supporting safer and more durable energy‐storage technologies for a wide range of applications. Despite significant advances in SPEs, several fundamental scientific and technological challenges must be addressed before their widespread implementation in practical solid‐state batteries. Future evaluation of SPEs should extend beyond conventional electrochemical measurements and postcycling surface observations toward standardized quantitative characterization of degradation processes. While scanning electron microscopy provides valuable information on surface morphology, it often cannot resolve the 3D evolution of lithium dendrites or internal failure pathways within solid electrolytes. Advanced characterization techniques, including X‐ray computed tomography, focused ion beam tomography, and synchrotron‐based 3D imaging, enable quantitative analysis of dendrite penetration depth, volume fraction, propagation pathways, and electrolyte deformation. For example, Yi et al. [334] demonstrated that 3D dendrite reconstruction using 3D tomographic neutron depth profiling with improved spatial resolution and compositional range provides substantially more reliable insight into lithium growth behavior than 2D postcycling observations, highlighting the need for quantitative evaluation protocols when comparing different solid electrolyte systems. Future studies should therefore adopt standardized testing conditions together with multidimensional characterization to enable meaningful comparisons across electrolyte chemistries and practical cell configurations.

### 10.1 Integrating Ion Transport, Mechanics, and Interfaces into Unified Electrolyte Design

Future electrolyte development should move beyond optimizing individual performance metrics such as ionic conductivity, lithium‐ion transference number, or mechanical modulus in isolation. Practical battery performance is governed by the coupled interactions among ion transport, mechanical deformation, interfacial stability, and electrochemical reactions during long‐term cycling. Developing multifunctional electrolytes that simultaneously satisfy these requirements remains one of the foremost challenges.

### 10.2 Understanding Dynamic Interfaces under Realistic Operating Conditions

Although considerable progress has been achieved in interface engineering, the polymer–electrode interface remains the primary source of performance degradation. Future research should focus on understanding the dynamic evolution of the SEI and CEI under electrochemical cycling through the combined use of *operando* characterization techniques and multiscale computational modelling. Stable, self‐adaptive interphases rather than static interfacial coatings are likely to be essential for long‐term cycling stability.

### 10.3 Establishing Predictive Structure–property Relationships

Recent advances in polymer chemistry, block copolymers, composite electrolytes, and single‐ion conductors have demonstrated that electrolyte performance is highly sensitive to molecular architecture and microstructure. However, quantitative relationships linking polymer morphology, ion coordination, transport pathways, and macroscopic battery performance remain poorly understood. Future studies should combine advanced characterization, atomistic simulations, and data‐driven modelling to establish predictive design principles for next‐generation SPEs.

### 10.4 Accelerating Translation from Laboratory Demonstrations to Practical Cells

Many promising electrolyte systems continue to be evaluated under idealized laboratory conditions. Greater emphasis should be placed on full‐cell testing using practical cathode loadings, limited lithium excess, realistic current densities, extended cycling, and transparent failure analysis. Standardized testing protocols and consistent reporting practices will improve reproducibility and enable meaningful comparisons across different electrolyte systems.

### 10.5 Leveraging Artificial Intelligence and Autonomous Materials Discovery

The rapid expansion of experimental and computational datasets provides new opportunities for artificial intelligence‐assisted electrolyte discovery. Integrating machine learning with high‐throughput experimentation, multiscale simulations, and automated synthesis platforms could substantially accelerate the identification of polymer chemistries, filler combinations, and interface designs with optimized electrochemical and mechanical performance.

## Author Contributions

**Mohan Raj Krishnan:** original manuscript writing and editing. **Chandra Sekhar Bongu** **:** original manuscript writing and editing. **Edreese Alsharaeh** **:** supervision, funding acquisition. All authors have read and agreed to the published version of the manuscript.

## Funding

This research was funded by Alfaisal University, grant number PIF‐726174.

## Conflicts of Interest

The authors declare no conflicts of interest.

## Data Availability

Experimental data will be available from the corresponding author on request.
